# Supplementary material for: Trophectoderm mechanics direct epiblast shape upon embryo implantation
Source: Cell Rep. 2021 Jan 19;34(3):108655. doi: 10.1016/j.celrep.2020.108655 (PMC7816124; doi:10.1016/j.celrep.2020.108655)
Supplement: Document S1. Figures S1–S6 [file mmc1.pdf]

Cell Reports, Volume *34*

Supplemental Information

Trophectoderm mechanics direct epiblast shape upon embryo implantation

Antonia Weberling and Magdalena Zernicka-Goetz

Supplementary

Supplementary Figure S1

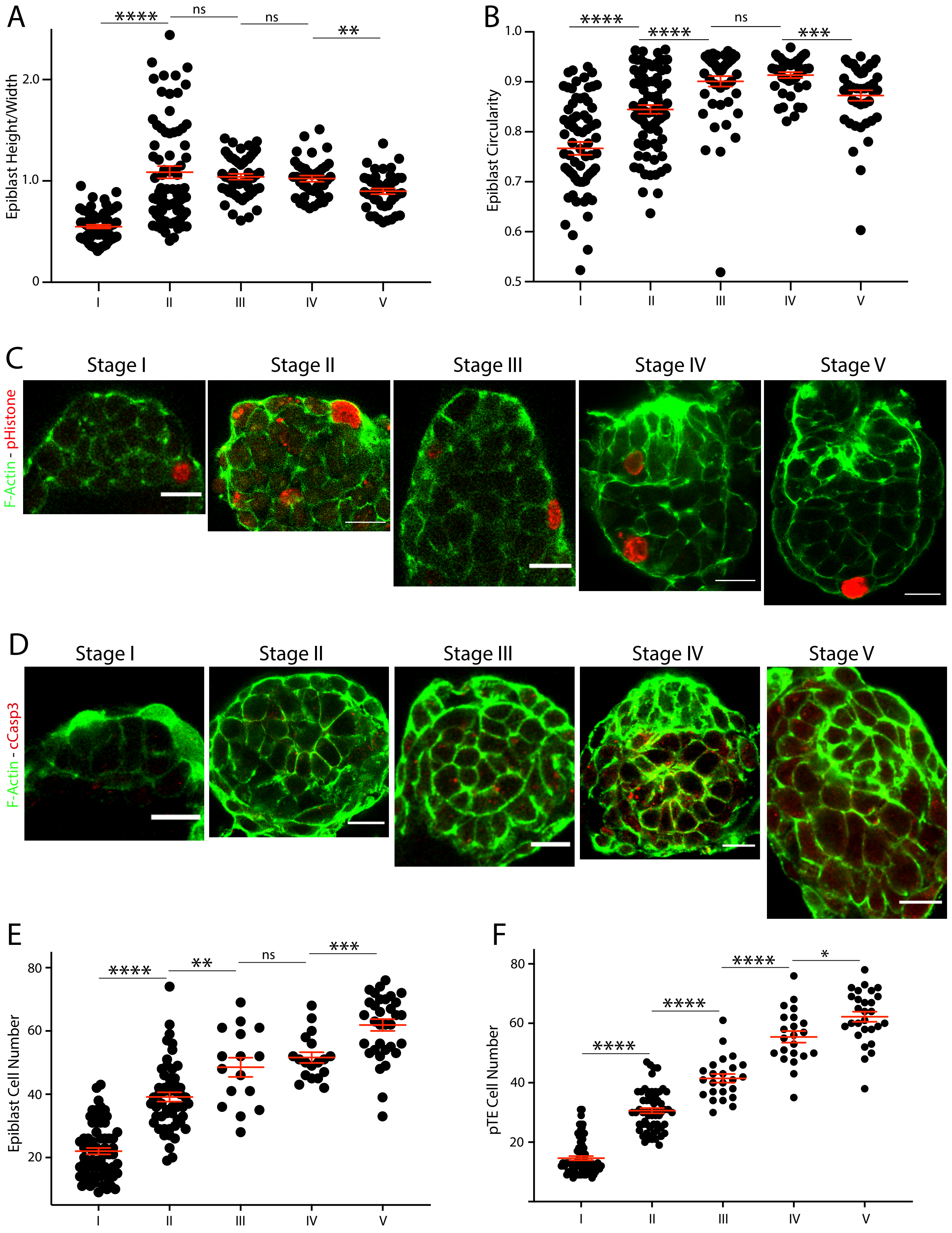

Figure S1. Epiblast and polar trophectoderm do not show polarised cell death or proliferation upon implantation. related to Figure 1 a. Quantitative analysis of epiblast aspect ratio (Height/Width) over time. Scatter plot, Mean±SEM (red). The aspect ratio increased from stage I-II significantly to then stay constant and only decrease slightly from stage IV to V. Analysis: unpaired student's t test. Stage I-II: $p<0.0001$, stage II-III: $p=0.5709$, stage III-IV 0.6591, stage IV-V: $p= 0.0029$. **b.** Quantification of epiblast Circularity over time. Scatter plot, Mean±SEM. The circularity increases first to drop after stage IV. N numbers as in (g). Analysis: unpaired student's t test. Stage I-II: p <0.0001, stage II-III: p <0.0001, stage III-IV 0.3371, stage IV-V: p 0.0012. **c.** IF analysis of pospho-Histone 3 over time. Embryos were stained for F-Actin (green) and phospho-Histone 3 (pHistone) (red). **d.** IF analysis of apoptosis at peri-implantation stages through staining for cleaved Caspase 3 (cCasp3) (red) and F-Actin (green). **e.** Quantitative analysis of epiblast cell numbers over time. The epiblast exhibits a high mitotic index throughout peri-implantation stages. Scatter plot, Mean±SEM (red). Analysis unpaired student's t-test: stage I-II: $p<0.0001$, stage II-III: $p=0.0041$, stage III-IV: $p=0.3726$, stage IV-V: $p=0.0007$. N numbers: stage I: 71, stage II: 53, stage III: 16, stage IV: 18, stage V: 34. **f.** Quantitative analysis of polar TE cell numbers over time. The polar TE also exhibits a high mitotic index throughout peri-implantation stages. Scatter plot, Mean±SEM (red). Analysis unpaired student's t-test: stage I-II: $p<0.0001$, stage II-III: $p<0.0001$, stage III-IV: $p<0.0001$, stage IV-V: $p=0.0114$. N numbers: stage I: 71, stage II: 61, stage III: 24, stage IV: 22, stage V: 28.

Supplementary Figure S2

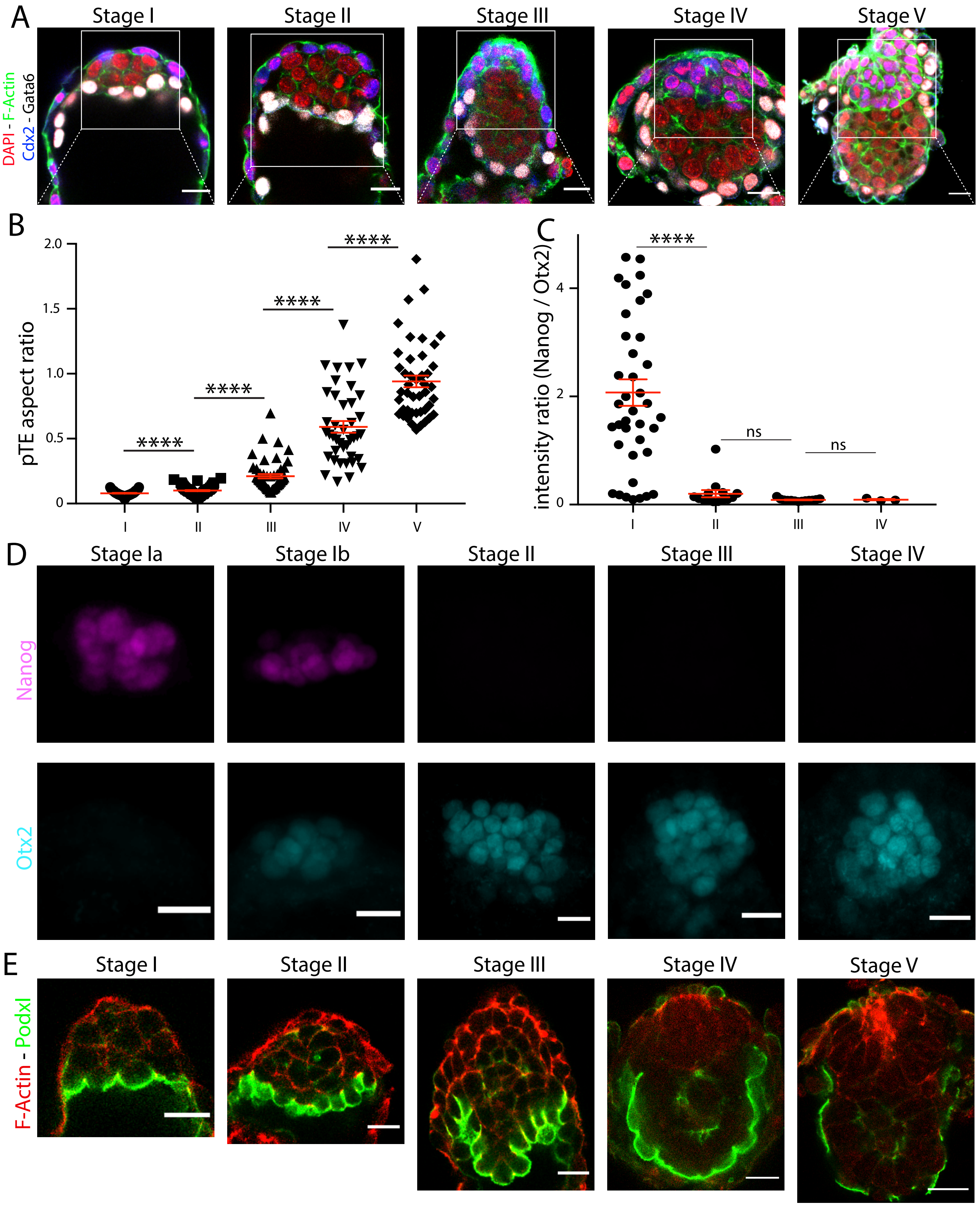

Figure S2. Dynamics of the Tissue interface from implantation to egg cylinder formation, related to Figure 2. a. Lineage staining of embryos fixed at sequential time points from implantation to egg cylinder formation. Full size samples of embryos shown and annotated in Figure 2A. Embryos stained for DAPI (red), Gata6 (white) Cdx2 (blue) and F-Actin (green). **b.** Quantitative analysis of the polar TE aspect ratio (polar TE average height/interface length) over time. Scatter plot, Mean±SEM (red). The aspect ratio increased exponentially. Analysis: unpaired student's t test. Stage I-II: $p<0.0001$, stage II-III: $p<0.0001$, stage III-IV<0.0001, stage IV-V: $p<0.0001$. **c.** Quantitative analysis of the differentiation status of the epiblast. Nanog mean grey value/Otx2 mean grey value. For each embryo, 3 measurements were obtained, the average for each embryo was plotted. The Otx2 expression became clearly upregulated following stage I. In stage I, two clusters are visible. Analysis: unpaired student's t test. Stage I-II: $p<0.0001$, stage II-III: p= 0.1120, stage III-IV 0.7389. **d.** z-projection (Average Intensity) of embryos fixed at consecutive stages following implantation. Nanog was downregulated during stage Ib. Otx2 became upregulated upon stage Ib. **e.** IF Analysis of epiblast polarisation marker PodxI over time. The epiblast becomes fully polarised at stage IV. PodxI (green), F-Actin (red). All scale bars 20µm.

Supplementary Figure S3

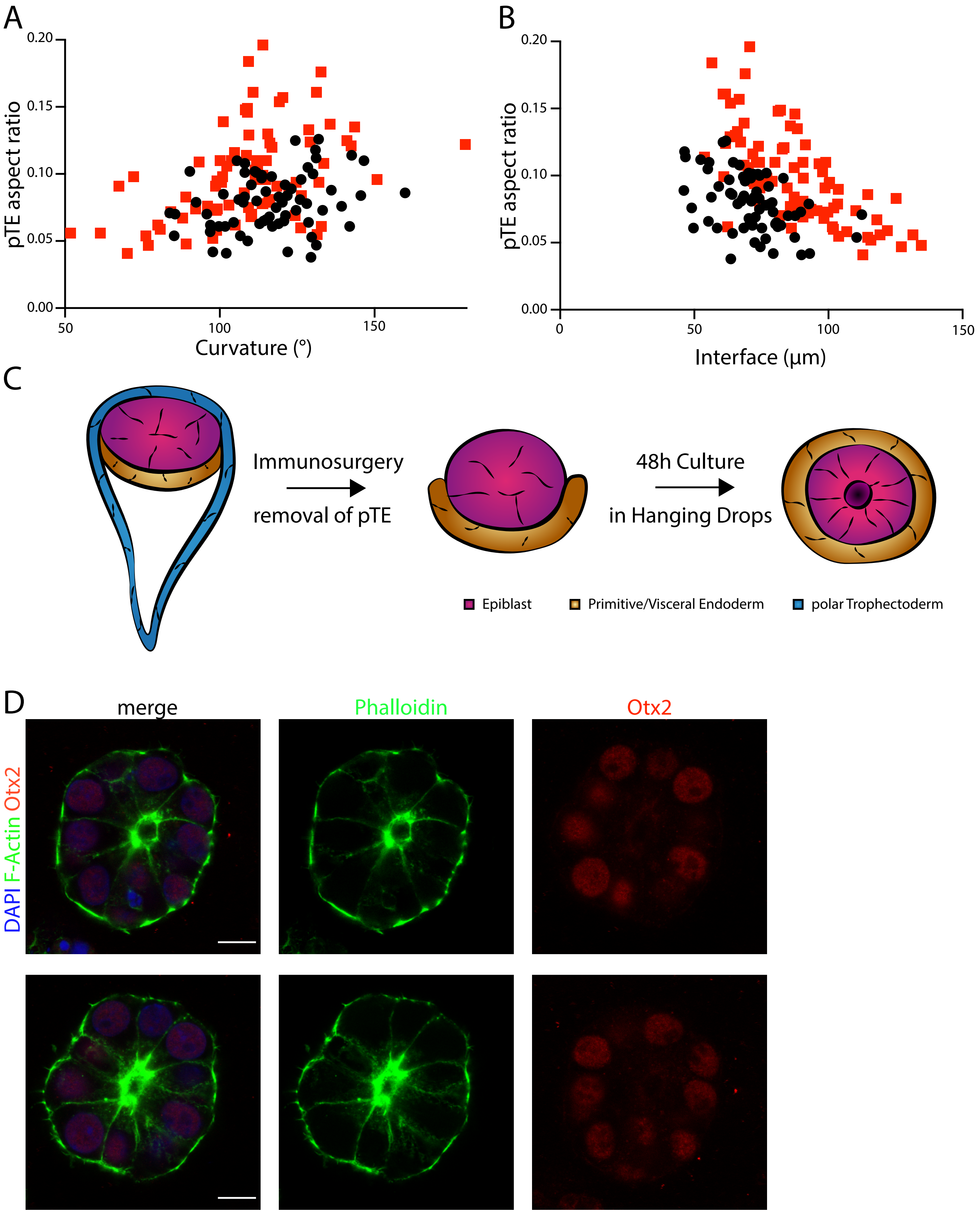

Figure S3. Correlation of polar trophectoderm with curvature and Interface length between stage I and stage II, related to Figure 3. a. Correlation of the polar TE aspect ratio versus the total curvature of the tissue interface for stage I (black) and stage II (red). It is visible, that the two timepoints strongly overly each other. **b.** Correlation of the polar TE aspect ratio versus the total interface length for stage I (black) and stage II (red). It is visible, that these show the opposite trend with an increase in interface length than the following stages (Figure 3B). **c.** Schematic of Immuno-surgery and the following culture. Through Immuno-surgery, the trophectoderm lineage (blue) is removed, the epiblast (red), covered on its' distal side with the primitive endoderm (brown), is cultured in hanging drops for 48h, during which it becomes spherical and opens a lumen while the primitive endoderm spreads to cover the entire epiblast. **d.** Examples of outlier mESC structures grown for 48h in differentiating conditions that exhibited low circularity (Figure 3F).

Supplementary Figure S4

A

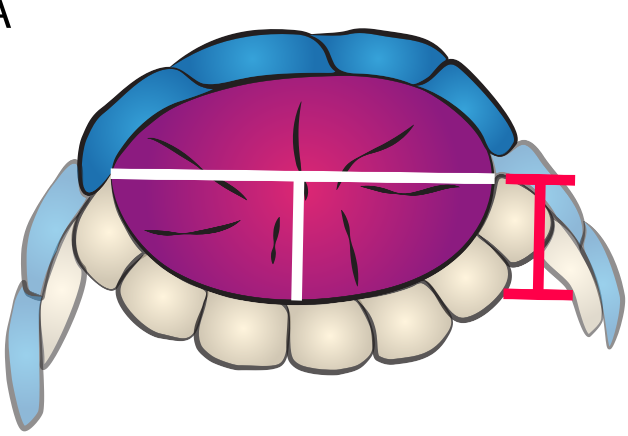

Figure S4. The Epiblast is pushed into the blastocoelic cavity upon implantation, related to Figure 4. a. Schematic of the measurement of the pushing distance. Polar trophectoderm (blue), epiblast (magenta-purple), primitive endoderm (beige). The distance was measured according to the white and red annotations.

Supplementary Figure S5

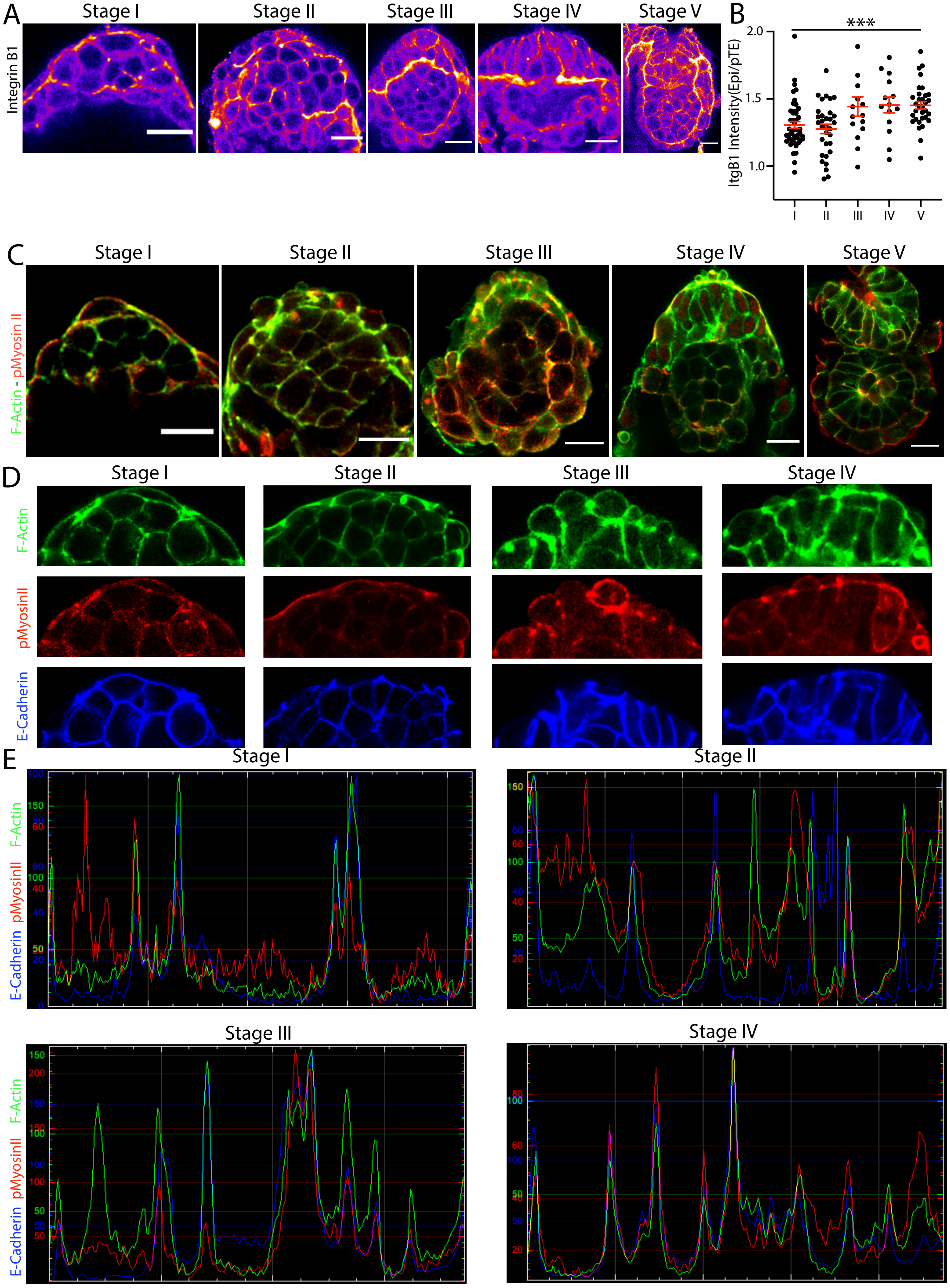

Figure S5. pMyosin II and F-Actin show strong correlation at the apical cell-cell junctions in the polar trophectoderm during epiblast cup shape formation, related to Figure 5. a. IF staining of mouse embryos at peri-implantation stages with basal marker Integrin β1. Fire-staining represents intensity of signal with purple being lowly expressed and yellow showing high signal intensity. It becomes visible, that the polar trophectoderm (polar TE) has a higher intensity of Integrin β1 than the epiblast. **b.** Quantitative Analysis of the intensity of Integrin β1 in epiblast versus polar TE over time. Scatter plot, Mean±SEM (red). Analysis stage I-V: unpaired students t-test: $p=0.0004$. The intensity increases significantly in the polar TE. N-numbers: stage I: 46, stage II: 32, stage III: 15, stage IV: 14, stage V: 32. **c.** Staining of F-Actin (green) and pMyosin II (red) in embryos fixed at consecutive timepoints upon implantation. Increased localisation of F-actin and pMyosin II becomes visible at the apical cell-cell junctions. From stage III to stage IV, a continuous actin cable could be observed. **d.** Zoom-in of Figure 5g, F-Actin (green), pMyosin II (red), E-Cadherin (blue). **e.** Zoom of Figure 5h: Merged Plot profiles of the apical surface of the polar TE in (c). A spline fit line was drawn with a thickness of 5µm. Plot profile determined through Fiji. Green resembles F-actin, red pMyosin-II, blue E-Cadherin. It is visible that from stage I to stage IV, the peaks of each marker begin to overlay. All scale bars 20µm.

Supplementary Figure S6

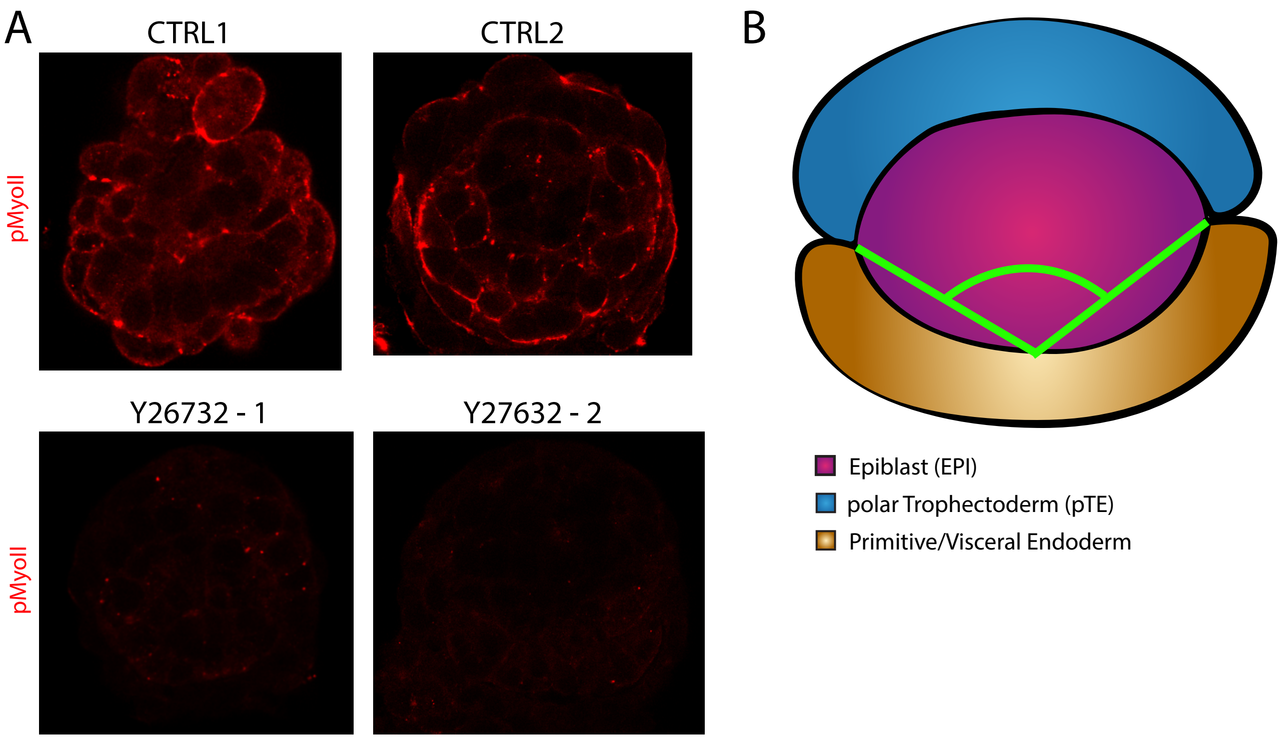

Figure S6: Mouse embryos mimic the human morphogenesis when exposed to Rock inhibitor, related to Figure 6. a. IF staining of pMyosin II channel of the embryos shown in Figure 6h. pMyosin II is lost after Rock inhibition. b. Schematic for quantification of primitive endoderm coverage angle quantified in Figure 6i. Epiblast in red, polar trophectoderm in blue, primitive endoderm in brown. The coverage angle measured is indicated in green.
