# Supplementary material for: Trophectoderm mechanics direct epiblast shape upon embryo implantation
Source: Cell Rep. 2021 Jan 19;34(3):108655. doi: 10.1016/j.celrep.2020.108655 (PMC7816124; doi:10.1016/j.celrep.2020.108655)
Supplement: Document S2. Article plus supplemental information [file mmc3.pdf]

Cell Reports

Article

Trophectoderm mechanics direct epiblast shape upon embryo implantation

Graphical Abstract

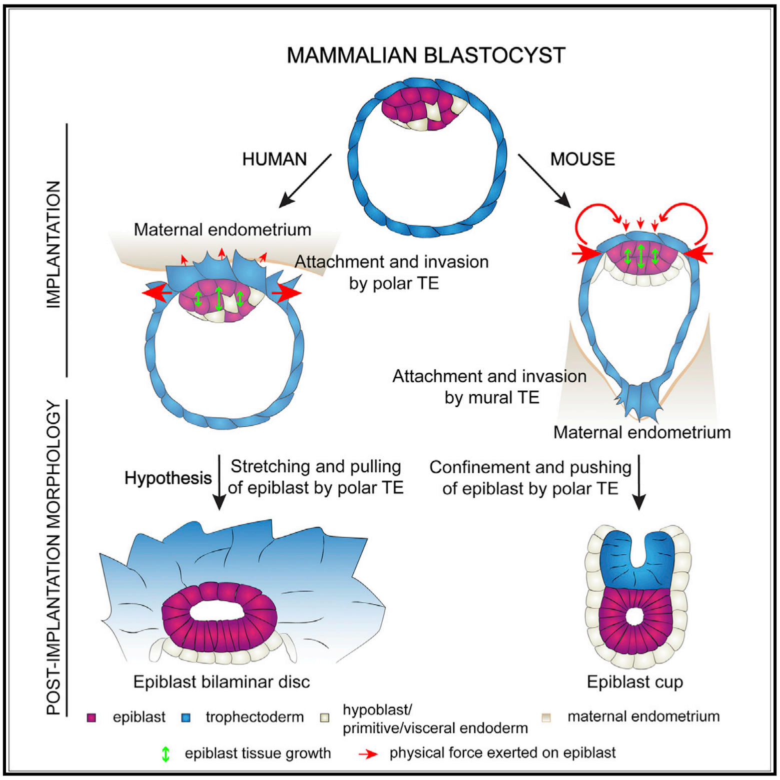

Authors

Antonia Weberling, Magdalena Zernicka-Goetz

Correspondence

mz205@cam.ac.uk

In Brief

Weberling and Zernicka-Goetz characterize the remodeling of the mouse epiblast upon implantation. Its transformation from oval to cup shaped is driven by the polar trophectoderm tissue. Comparative analyses with human embryos and inhibitor treatments suggest the polar trophectoderm as an evolutionarily conserved regulator of epiblast shape upon implantation.

Highlights

- Mouse epiblast remodeling from blastocyst to egg cylinder is achieved in five stages
- Epiblast remodeling upon implantation is not inherent to the embryonic lineage
- The polar trophectoderm mediates epiblast shape acquisition
- Epiblast shape regulation by the polar trophectoderm appears conserved in evolution

Weberling & Zernicka-Goetz, 2021, Cell Reports *34*, 108655
January 19, 2021 © 2021 The Authors.
https://doi.org/10.1016/j.celrep.2020.108655

CellPress

Cell Reports

CellPress
OPEN ACCESS

Article

Trophectoderm mechanics direct epiblast shape upon embryo implantation

Antonia Weberling[1] and Magdalena Zernicka-Goetz[1,2,3,*]
[1]Mammalian Embryo and Stem Cell Group, University of Cambridge, Department of Physiology, Development and Neuroscience, Downing Street, Cambridge CB2 3DY, UK
[2]Plasticity and Self-Organization Group, California Institute of Technology, Division of Biology and Biological Engineering, 1200 E. California Boulevard, Pasadena, CA 91125, USA
[3]Lead contact
*Correspondence: mz205@cam.ac.uk
https://doi.org/10.1016/j.celrep.2020.108655

SUMMARY

Implantation is a hallmark of mammalian embryogenesis during which embryos establish their contacts with the maternal endometrium, remodel, and undertake growth and differentiation. The mechanisms and sequence of events through which embryos change their shape during this transition are largely unexplored. Here, we show that the first extraembryonic lineage, the polar trophectoderm, is the key regulator for remodeling the embryonic epiblast. Loss of its function after immuno-surgery or inhibitor treatments prevents the epiblast shape transitions. In the mouse, the polar trophectoderm exerts physical force upon the epiblast, causing it to transform from an oval into a cup shape. In human embryos, the polar trophectoderm behaves in the opposite manner, exerting a stretching force. By mimicking this stretching behavior in mouse embryogenesis, we could direct the epiblast to adopt the disc-like shape characteristic of human embryos at this stage. Thus, the polar trophectoderm acts as a conserved regulator of epiblast shape.

INTRODUCTION

During implantation, the mammalian embryo comprises three lineages: the extra-embryonic trophectoderm (TE), the primitive endoderm, and the embryonic epiblast. The TE forms a hollow cyst, enclosing the proximal side of the epiblast with its polar part and the blastocoelic cavity with its mural part. In mouse embryos, the epiblast is covered on its distal side by the primitive endoderm, whereas in human embryos, the second lineage segregation has not yet been completed at this stage. In the mouse, implantation takes place at embryonic day (E)4.5 and is mediated by the mural TE followed by a series of remodeling events that lead to the formation of the egg cylinder, a characteristic of rodent embryos (Molè et al., 2020; Smith, 1980). The establishment of a tissue boundary between polar and mural TE leads to the transformation of the polar TE from a squamous to a thick pseudostratified epithelium due to an increased cell proliferation rate and high contractility (Copp, 1978, 1979; Christodoulou et al., 2019). Invagination of the polar TE via apical constriction pushes the epiblast into the blastocoelic cavity, giving rise to the cylindrical morphology of the post-implantation embryo (Christodoulou et al., 2019). Human embryo implantation is mediated by the polar TE instead, and its post-implantation morphogenesis diverges drastically from the mouse, leading to the formation of a bilaminar disc-shaped epiblast as opposed to the egg cylinder (Hertig et al., 1956; Molè et al., 2020).

Generation of forces at the cellular level, their integration and propagation across tissues drives tissue morphogenesis (Heer and Martin, 2017; Martin et al., 2010; Pinheiro and Bellaïche, 2018). For this, forces must first be built up in individual cells through actomyosin networks that generate contractility and, thereby, tension in the cell cortex (Cartagena-Rivera et al., 2016; Chugh et al., 2017; Svitkina, 2020). The actomyosin-rich cortex is bound to the cell membrane by the α-catenin-β-catenin complex that regulates cytoskeletal organization through α-catenin-E-cadherin interactions (Nelson, 2008; Yamada et al., 2005). E-cadherin forms adherens junctions and allows propagation of tension throughout tissue. Increased tension results in the growth and stabilization of the junctions to ensure tissue integrity. Thus, E-cadherin serves as a mechano-sensor (Buckley et al., 2014; le Duc et al., 2010; Martin et al., 2010; Pinheiro and Bellaïche, 2018).

Upon implantation, the epiblast remodels on the cellular level to form an epithelium (Bedzhov and Zernicka-Goetz, 2014; Wallingford et al., 2013). This remodeling and the parallel cell polarization are dependent on extracellular matrix (ECM) components. Upon blastocyst formation at E3.5, the mural TE begins to deposit ECM components along its basal side, giving rise to Reichert's membrane. At E4.5, this membrane consists of thick, multi-layered ECM forming a continuous structure with a second basement membrane (BM) established predominantly by the primitive endoderm and deposited between this tissue and the epiblast (Salamat et al., 1995). Formation of both membranes is critical for embryo survival (Miner et al., 2004; Smyth et al., 1999) and epiblast morphogenesis (Bedzhov and Zernicka-Goetz, 2014; Fässler and Meyer, 1995). However, whether and

This is an open access article under the CC BY license (http://creativecommons.org/licenses/by/4.0/).

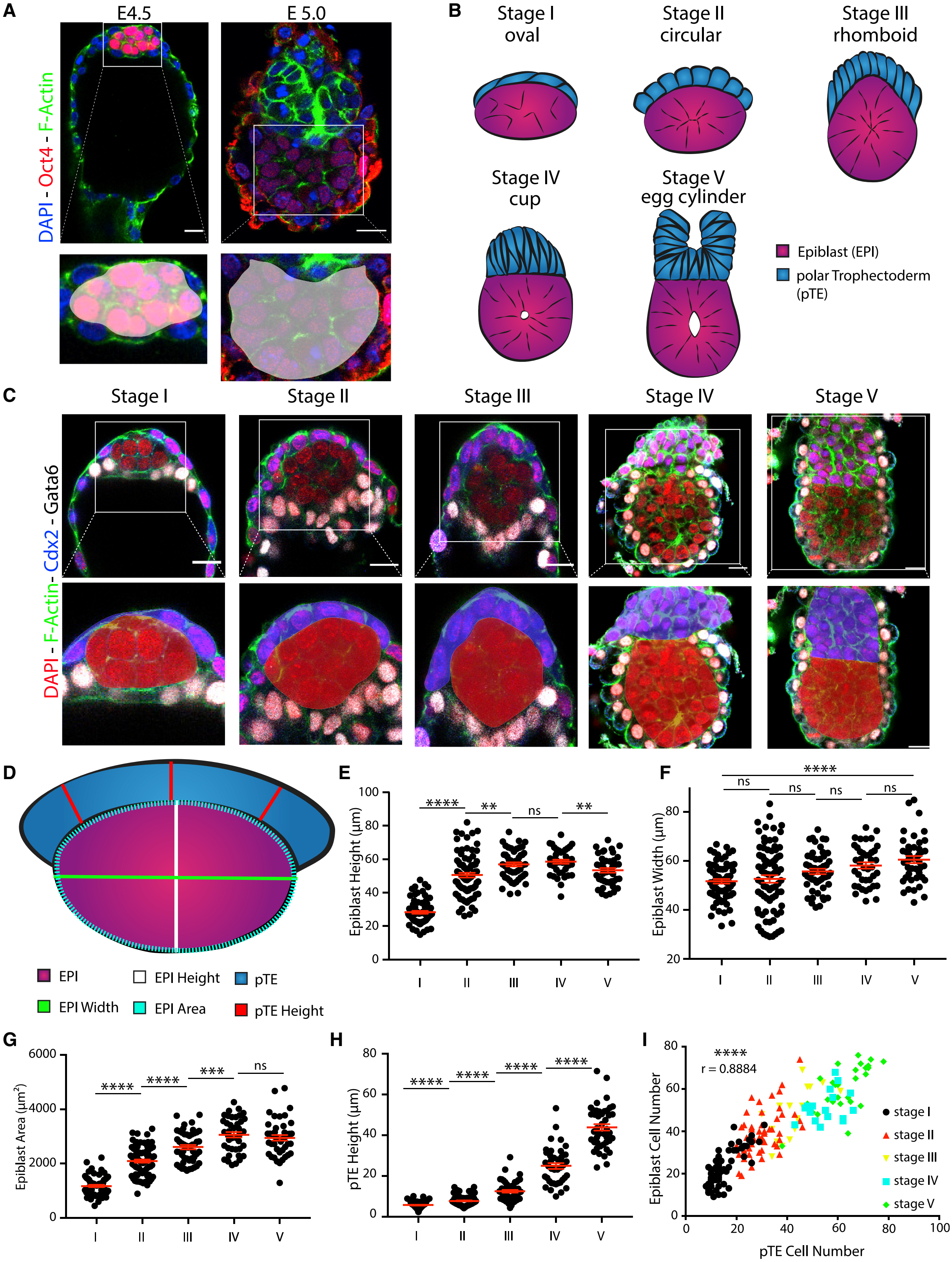

(legend on next page)

how the ECM could affect the acquisition of tissue shape during the implantation stages are currently unknown.

Here, we studied the tissue remodeling events that drive peri-implantation morphogenesis of the epiblast in mouse and human embryos. We found that acquisition of the characteristic cup shape in mice is determined by increasing contractility and tension in the polar TE, which generates a physical force to push the epiblast into its post-implantation configuration. The polar TE of human embryos acts in the opposite way, exerting a stretching force on the epiblast, leading it to adopt a disc-like structure. The polar TE, therefore, appears to be an evolutionarily conserved regulator of epiblast shape upon implantation.

RESULTS

The steps of mouse embryogenesis during transition from blastocyst to egg cylinder

During the transition from pre- to post-implantation, the mouse epiblast transforms from an oval to a cup-shaped morphology (Figure 1A). To investigate the mechanism underlying this transition, we first carried out a quantitative analysis of epiblast shape at consecutive time points from implantation to egg-cylinder formation. We found that the epiblast progressed through 5 distinct states (Figure 1B). Initially, it exhibited an oval shape with its long axis parallel to the polar TE, which formed a thin layer of squamous cells (Figure 1C, stage I). However, 6 h after the initiation of implantation, the epiblast became spherical, doubling its total height but retaining a constant width (Figures 1D–1G, stages I and II; Figures S1A and S1B). During the same time period, the polar TE increased in height (Figure 1C, stage II, and 1H, stages I and II). Strikingly, the epiblast continued to grow in height but not in width during the next 5 h of development, until it acquired a highly angled rhomboid shape (Figures 1C–1G; Figures S1A and S1B, stage III), while the polar TE increased further in height (Figure 1H, stages II and III). The next developmental stage was characterized 5 h later by a pronounced rearrangement of the epiblast from the rhomboid to a cup shape, whereupon it came to share a straight tissue interface with the polar TE, which generated a dome-like structure on top of the epiblast (Figure 1C, stage IV). During this rearrangement, the epiblast remained constant in both height and width (Figures 1E and 1F, stages III and IV) but exhibited continuous growth in total area while retaining a spherical shape (Figure 1G; Figure S1B, stages III and IV). In contrast, the polar TE continued to increase exponentially in height (Figure 1H, stages III and IV). The embryo completed its transformation 1–2 h after cup-shape acquisition by folding of the polar TE through apical constriction (Christodoulou et al., 2019) and the epiblast fully acquiring its cup shape, giving rise to the post-implantation egg cylinder (Figure 1C, stage V). Subsequently, the epiblast gradually lost its spherical shape to become more cuboid (Figures 1D-1G; Figure S1B, stages IV and V). To understand whether these shape changes in the different lineages could be accounted for by localized cell division or death, we determined the distribution of phospho-histone 3 and cleaved caspase-3, respectively (Figures S1C and S1D) and quantified the cell numbers (Figures S1E and S1F). Both lineages exhibited a parallel increase in cell number throughout the implantation period, but we could not observe any specific localized cell proliferation or death (Figure 1I; Figures S1C and S1D). These results indicate that the epiblast and the polar TE undergo dynamic remodeling after implantation with the epiblast evolving through several distinct spatial configurations before reaching cup shape, which cannot be accounted for by localized tissue proliferation or cell death.

The epiblast and polar trophectoderm interface remodels during transition from blastocyst to egg cylinder

To investigate whether the remodeling of the epiblast and polar TE were interconnected, we focused on the tissue interface (Figure 2A; Figure S2A) and quantified total length, diameter, and curvature angle (Figure 2B). Upon implantation, the proximal side of the oval epiblast was covered by the polar TE (Figure 2A, stage I; Figure S2A, stage I). Length, diameter, and the total

Figure 1. Sequence of remodeling steps of epiblast and polar trophectoderm tissues upon implantation

(A) E4.5 implanting blastocyst and E5.0 early egg cylinder. Staining: DAPI (blue), F-actin (green), and Oct4 (red). Oct4 is expressed in the epiblast tissue. Zoom-in on the epiblast tissue highlights shapes of the epiblast upon implantation (oval) and post-implantation (cup).

(B) Schematic of the epiblast (pink) and polar TE (blue) lineages from implantation to egg-cylinder formation.

(C) Lineage staining of embryos fixed at sequential time points from implantation to egg-cylinder formation (E4.5–5.0). Top row: embryos stained for Gata6 (white) and Cdx2 (blue) to distinguish primitive endoderm and polar TE lineages, respectively. Staining: DAPI (red) and F-actin (green). This allows analysis of epiblast and polar TE tissue shapes. Bottom row: zoom-in on epiblast and polar TE lineages, with polar TE highlighted in blue and the epiblast in red.

(D) Schematic to illustrate measurements taken for quantitative analysis. Polar TE is indicated in blue, and epiblast is indicated in pink. Measurements were taken in plane of maximum tissue area for both lineages. Epiblast height (white) and width (green) were measured through the center of the epiblast. Epiblast area (green dotted line) was measured for the maximum area. Polar TE height (red) was measured at three points, and the average for each embryo was analyzed.

(E) Quantification of epiblast height (in microns) over time. Scatterplot, mean ± SEM. The epiblast height changes significantly over time. Stage I, $n = 69$; stage II, $n = 81$; stage III, $n = 51$; stage IV, $n = 40$; stage V, $n = 43$. Analysis, unpaired Student's t test: stages I–II, $p < 0.0001$; II–III, $p = 0.0020$; III–IV, $p = 0.3530$; IV–V, $p = 0.0059$.

(F) Quantification of epiblast width over time. Scatterplot, mean ± SEM. Epiblast width increases slightly. Stage I, $n = 68$; stage II, $n = 81$; stage III, $n = 51$; stage IV, $n = 40$; stage V, $n = 43$. Analysis, unpaired Student's t test: stages I–II, $p = 0.6192$; II–III, $p = 0.1559$; III–IV, $p = 0.1523$; IV–V, $p = 0.2277$; I–V, $p < 0.0001$.

(G) Quantification of epiblast area over time. Scatterplot, mean ± SEM. Area increases significantly over time. Stage I, $n = 69$; stage II, $n = 81$; stage III, $n = 51$; stage IV, $n = 40$; stage V, $n = 44$. Analysis, unpaired Student's t test: stages I–II, $p < 0.0001$; II–III, $p < 0.0001$; III–IV, $p = 0.0005$; IV–V, $p = 0.4385$.

(H) Quantification of polar TE height over time. Scatterplot, mean ± SEM. Height of polar TE increases exponentially over time. Stage I, $n = 69$; stage II, $n = 82$; stage III, $n = 51$; stage IV, $n = 40$; stage V, $n = 44$. Analysis, unpaired Student's t test: stages I–II, $p < 0.0001$; II–III, $p < 0.0001$; III–IV, $p < 0.0001$; IV–V, $p < 0.0001$. Scale bars, 20 μm.

(I) Analysis of epiblast and polar TE cell numbers over time. Stages I: black, II: red, III: yellow, IV: blue, V: green. The growth in cell numbers is highly correlated; Pearson $r = 0.8884$, $p < 0.0001$. Stage I, $n = 62$; stage II, $n = 51$; stage III, $n = 16$; stage IV, $n = 17$; stage V, $n = 28$.

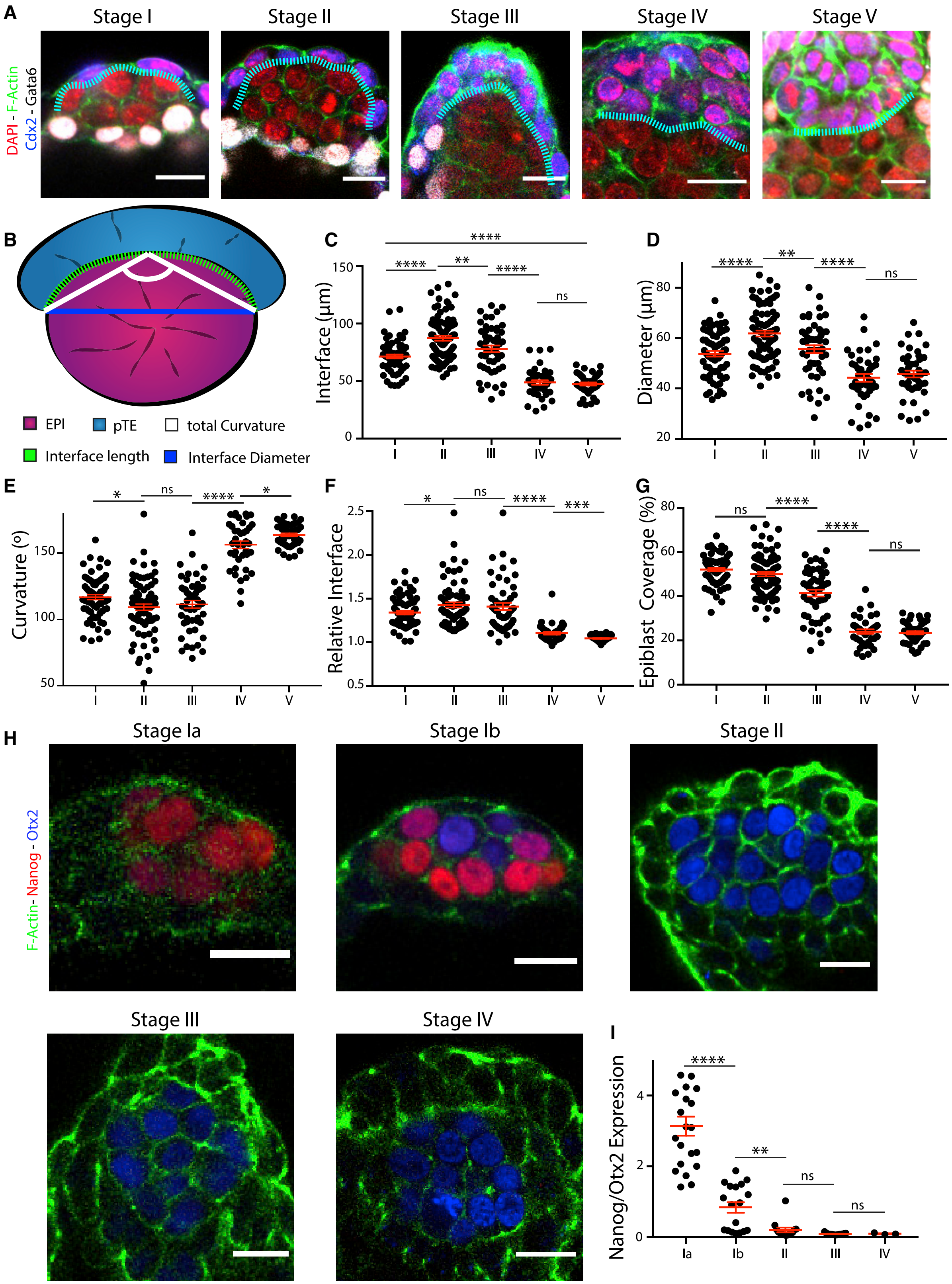

(legend on next page)

curvature of the interface increased from stage I to stage II (Figures 2C–2E, stages I and II). However, when the epiblast acquired a rhomboid shape (stage III), both interface length and diameter dropped while the curvature remained, suggesting an increase in relative curvature as the interface had become smaller (Figures 2C–2E, stages II and III). Concurrent with the remodeling of the epiblast into the cup, the polar TE-epiblast interface transformed from being highly curved into a straight line (Figures 2A–2E, stages III and IV), indicating that the major tissue rearrangement takes place between stage III and stage IV. At stage V, the interface curvature decreased further to almost 180° (Figures 2C–2E, stage V). At the same time, the relative interface (interface diameter/interface total length) approached 1 (Figure 2F). Consequently, we found that the polar TE covered 50% of the epiblast at stages I and II, which decreased to 25% after cup-shape acquisition (stages IV and V) (Figure 2G) while increasing exponentially in aspect ratio (Figure S2B). Together, this suggests that the epiblast minimizes its surface presented to the polar TE during egg-cylinder formation in a transition from a highly curved to a flat interface.

Alongside tissue architecture changes, the transition from pre- to post-implantation is marked by the epiblast exiting the naive toward the primed pluripotent state (Nichols and Smith, 2009). To correlate the pluripotency state to the tissue shape changes, we analyzed the expression of markers for the naive state, Nanog, and the primed state, Otx2. Nanog expression became downregulated during stage I, which we, therefore, further subdivided to represent embryos just before implantation and those that initiated implantation based on the morphology of their mural TE. Before implantation, embryos exhibited high levels of Nanog in all epiblast cells and no expression of the Otx2. Upon implantation, 50% of epiblasts showed upregulation of Otx2 and downregulation of Nanog. Nanog expression was completely abolished by stage II (Figures 2H and 2I; Figures S2C and S2D), indicating that exit from naive pluripotency is completed before epiblast remodeling. To correlate tissue shape changes to polarization on the single-cell level, we analyzed the localization of the apical marker Podxl. Podxl was expressed as early as stage II in non-focal patches that became localized to focal points by stage III and coalesced into a single lumen by stages IV and V (Figure S2E). This illustrates that the progression of epiblast polarization takes place in parallel to tissue remodeling but does not precede it.

The polar trophectoderm drives remodeling of the epiblast

To correlate tissue interface changes with the remodeling of the polar TE, we analyzed the total curvature and the interface length in relation to the polar TE aspect ratio (polar TE height/total interface length). We found strong correlations for both total curvature and interface length with the polar TE aspect ratio from stages II–V (Figures 3A and 3B), whereas from stages I and II, embryos exhibited no such correlation or even the opposite trend (Figures S3A and S3B). These observations suggest that the polar TE is the first tissue to be remodeled; therefore, we hypothesized that its remodeling might be driving the shape changes in the epiblast.

To test this, we removed the TE by immuno-surgery, incubating implanting blastocysts in anti-mouse serum and then complement serum. The TE was removed through pipetting, and embryos were placed in hanging drops of medium to prevent attachment to the dish and thus avoid epiblast deformation (Figure 3C; Figure S3C). The epiblast of embryos lacking the TE failed to remodel into a cup and displayed a symmetrical, circular shape (Figure 3D) in contrast to embryos from the control group, which became cup shaped. To confirm that both manipulated embryos and controls exited naive pluripotency, we analyzed the expression of Otx2. Both groups showed high Otx2 expression and had a re-arranged monolayered epithelium surrounding a single constriction point, thus having successfully exited the

Figure 2. The dynamics of the tissue interface suggest force transmission of the polar trophectoderm toward the epiblast

(A) Lineage staining of embryos fixed at consecutive time points from implantation to egg-cylinder formation. Staining: DAPI (blue), Gata6 (white), Cdx2 (blue), and F-actin (green). Tissue interface between epiblast and polar TE defined through F-actin (white dotted line).

(B) Schematic of quantifications carried out. Polar TE (blue) and epiblast (pink). Interface was analyzed for the following parameters: total length of interface (green dotted line), interface diameter (blue), and total curvature angle (white).

(C) Quantitative analysis of tissue interface length over time. Scatterplot, mean ± SEM. Interface length increased and then dropped significantly. Stage I, $n = 68$; stage II, $n = 81$; stage III, $n = 51$; stage IV, $n = 40$; stage V, $n = 44$. Analysis, unpaired Student's t test: stages I–II, $p < 0.0001$; II–III, $p = 0.0081$; III–IV, $p < 0.0001$; IV–V, $p = 0.5749$.

(D) Quantitative analysis of diameter of tissue interface over time. Scatterplot, mean ± SEM. Diameter increased and then decreased to a steady state. Stage I, $n = 68$; stage II, $n = 81$; stage III, $n = 51$; stage IV, $n = 40$; stage V, $n = 44$. Analysis, unpaired Student's t test: stages I–II, $p < 0.0001$; II–III, $p = 0.0014$; III–IV, $p < 0.0001$; IV–V, $p = 0.4693$.

(E) Quantitative analysis of the total curvature of interface. Scatterplot, mean ± SEM. Curvature first dropped to then vastly increase going against 180°. Stage I, $n = 68$; stage II, $n = 81$; stage III, $n = 51$; stage IV, $n = 40$; stage V, $n = 44$. Analysis, unpaired Student's t test: stages I–II, $p = 0.0152$; II–III, $p = 0.5655$, III–IV, $p < 0.0001$, IV–V, $p = 0.0136$.

(F) Quantitative analysis of the relative interface (total length/diameter) over time. Scatterplot, mean ± SEM. Relative interface first increased from ~1.4 to ~1.45 to then go against 1. The ns are the same as in (C). Analysis, unpaired Student's t test: stages I–II, $p = 0.0229$; II–III, $p = 0.7226$; III–IV, $p < 0.0001$; IV–V, $p = 0.0005$.

(G) Quantitative analysis of epiblast coverage by the polar TE (total perimeter/length of interface) over time. Scatterplot, mean ± SEM. EPIBLAST was covered up to 50% by polar TE; this decreased to about 25% after cup formation. Stage I, $n = 68$; stage II, $n = 81$; stage III, $n = 51$; stage IV, $n = 40$; stage V, $n = 44$. Analysis, unpaired Student's t test: stages I–II, $p = 0.1128$; II–III, $p < 0.0001$; III–IV, $p < 0.0001$; IV–V: $p = 0.6305$.

(H) Staining of exit from naive pluripotency over time. Nanog (red) expressed only at stage I, and primed pluripotency marker, Otx2 (blue), from stage I onward and then steadily upregulated. F-actin (green) allows staging of the embryos.

(I) Quantitative analysis of expression dynamics of Nanog and Otx2. Mean gray value measured at 3 different z-positions per embryo. Mean of the ratio Nanog/Otx2 plotted. Nanog expression was lost already during stage I. Stage Ia, $n = 21$; stage Ib, $n = 18$; stage II, $n = 14$; stage III, $n = 13$; stage IV, $n = 3$. Scatterplot, mean ± SEM. Analysis, unpaired Student's t test: stages Ia–Ib, $p < 0.0001$; Ib–II, $p = 0.0015$; II–III, $p = 0.1120$; III–IV, $p = 0.7389$.

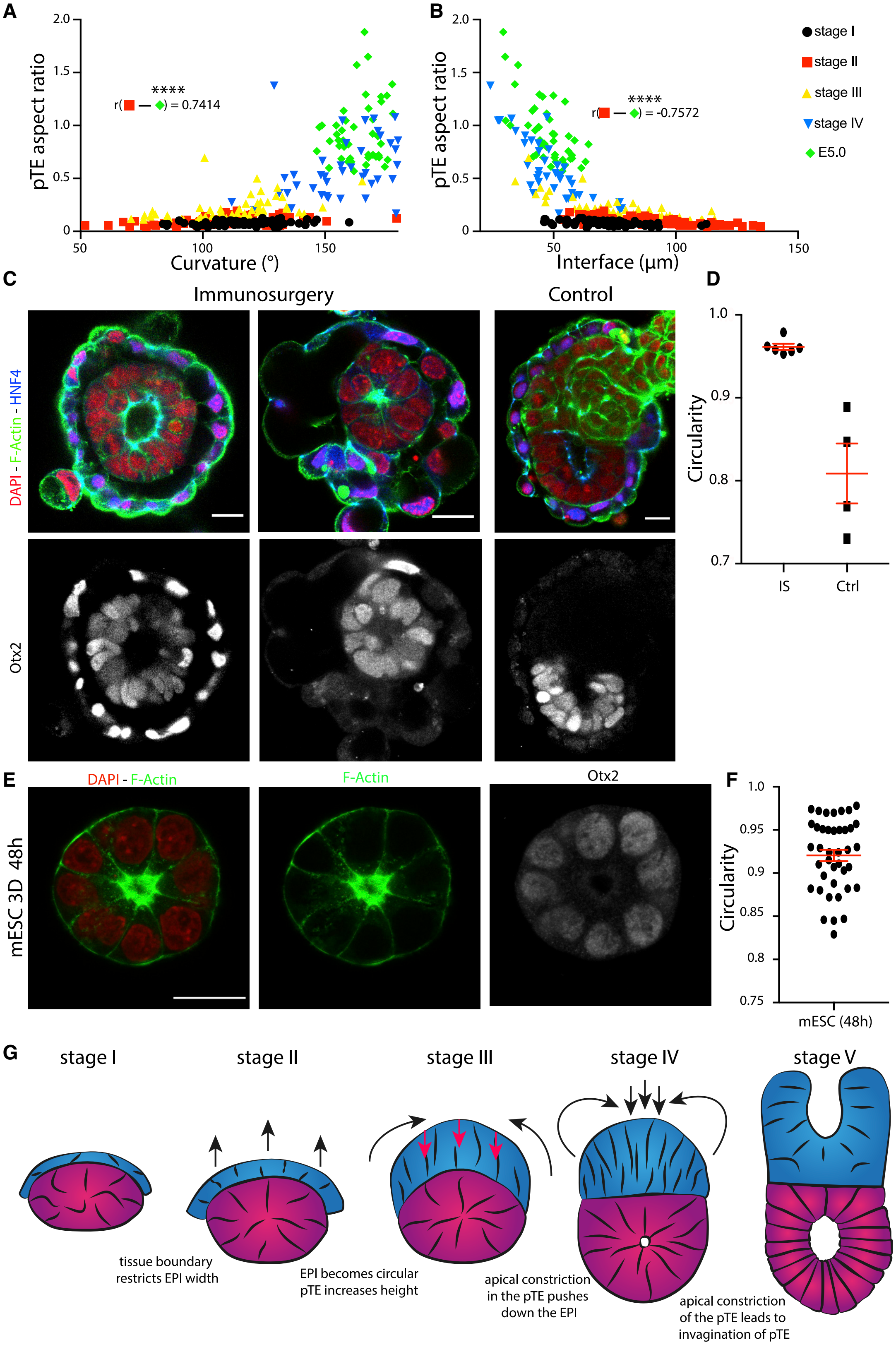

(legend on next page)

naive state. These results suggest that the polar TE is required for the epiblast shape transition.

To gain further insight on whether epiblast remodeling is dependent on the TE and not an inherent capacity of the epiblast, we utilized mouse embryonic stem cells (mESCs) to model the developing epiblast *in vitro* (Bedzhov and Zernicka-Goetz, 2014). We cultured mESCs in differentiating conditions in 3D Matrigel and found that, after 48 h of culture, the structures had become spherical, polarized around a focal lumen, and upregulated Otx2 expression (Figures 3E and 3F), in contrast to intact embryos. Even through few outliers exhibited low circularity, none acquired a cup shape (Figure S3D). These results suggest that cup-shape acquisition is not an inherent capacity of the epiblast during its differentiation process but that it depends on interaction with the polar TE (Figure 3G).

Differential deposition of the ECM may underline cup-shape acquisition

Our initial observation that epiblast width remains constant through remodeling led us to hypothesize that its horizontal expansion, perpendicular to the proximo-distal axis of the embryo, was restricted (Figure 1F). A potential, spatially restrictive scaffold could be introduced by the ECM as deletion of laminin, one of the main components of the ECM, leads to a failure of egg-cylinder formation (Miner et al., 2004; Smyth et al., 1999). We therefore decided to investigate the distribution of laminin, as an ECM marker, at implantation.

Initially, laminin was localized peri-cellularly within the primitive endoderm but had not yet formed a continuous BM along the distal tip of the epiblast (Figures 4A and 4B). At the same time, the ECM at the border of Reichert's membrane constituted a thick ring structure around the epiblast. In the following stages, a thin laminin-positive BM was established between epiblast and primitive endoderm while the border to Reichert's membrane continued to show the highest intensity of laminin deposition. After egg-cylinder formation, the BM uniformly surrounded the epiblast. Quantitative analysis of laminin localization revealed a continuous and highly significant increase in the intensity ratio of the BM relative to the border to Reichert's membrane from stages I–III, which stayed constant during the following stages (Figures 4C and 4D). This result led us to the hypothesis that the edge of the Reichert's membrane could introduce spatial constraint toward the epiblast, which would prohibit horizontal, but not vertical, growth and movement of the epiblast. To understand whether the maturation of the BM into a continuous layer could introduce a similar restriction vertically, we analyzed the distance through which the epiblast becomes pushed into the blastocoelic cavity (Figure S4A). Even though a continuous layer of ECM became established, the pushing distance continuously increased, suggesting that the thin BM may not be able to exert a restrictive force similar to that of Reichert's membrane (Figure 4E). These results suggest that the edge of Reichert's membrane could establish a tight ring around the epiblast, prohibiting horizontal growth leading to cup-shape acquisition.

Polar trophectoderm tension increases during epiblast remodeling

The dynamic behavior of the tissue interface between both lineages led us to hypothesize that differential tissue tension and contractility could result in the mechanical force required to drive the epiblast into its cup shape. To establish whether this could be the case, we examined the localization and total tissue expression levels of E-cadherin, F-actin, phosphorylated non-muscle myosin II (pMyosin-II), and integrin β1 (Ciobanasu et al., 2013; Sun et al., 2016) as a readout for tissue contractility and tension (Figures 5A–5C; Figure S5A) in embryos at consecutive stages upon implantation and measured the total intensity in epiblast versus polar trophectoderm (Figure 5D). Strikingly, we found that E-cadherin, F-actin, integrin β1, and pMyosin-II showed similar tissue intensities in the polar TE and the epiblast at stage I (Figures 5A–5F, stage I; Figures S5A and S5B, stage I). However, during subsequent development, the E-cadherin levels increased in the polar TE (Figures 5A and 5E, stage II). The same was true for F-actin (Figures 5B and 5F, stage II), which became localized specifically to the apical junctions within the polar TE, pMyosin-II (Figure 5C, stage II), and integrin β1 (Figures S5A and S5B, stage II). This trend continued up to stage IV for E-cadherin and then became constant (Figures 5A and 5E, stages III and IV). F-actin exhibited increasing intensity in the polar TE throughout development (Figures 5B and 5F, stages III and V), whereas pMyosin-II levels decreased slightly upon formation of the egg cylinder in the polar TE (Figure 5C, stages III and IV). Integrin β1 exhibited increasing expression levels in the polar TE until stage III and then remained constant (Figures S5A and S5B). These results suggest that contractility and tension increase within the polar TE compared to the epiblast during early post-implantation morphogenesis and that differential contractility between the epiblast and polar TE could be an underlying reason for cup-shape acquisition.

Figure 3. The polar trophectoderm induces cup-shape formation of the epiblast

(A) Correlation analysis of polar TE aspect ratio (total height/length of interface) with curvature. Stages II–V had a strong positive correlation. Stage I, n = 68; stage II, n = 81; stage III n = 51; stage IV, n = 40; stage V = 44. Analysis: r = 0.7414, $p < 0.0001$. Black, stage I; red, stage II; yellow, stage III; blue, stage IV; green, stage V.

(B) Correlation analysis of polar TE aspect ratio with the length of the tissue interface. Strong anti-correlation of stages II–V. Stage I, n = 68; stage II, n = 81; stage III, n = 51; stage IV, n = 40; stage V, n = 44. Analysis: r = −0.7572, $p < 0.0001$.

(C) Staining of E4.5 embryo cultured for 48 h in hanging drops after immuno-surgery (left and middle columns) and control (right column). Embryos stained for DAPI (red), F-actin (green), and HNF4alpha (blue, top row); Otx2 in the bottom row.

(D) Quantification of the circularity of the epiblast after immuno-surgery and hanging drop culture. For treated embryos (IS), only those were analyzed where the full TE tissue could be removed. For controls (Ctrl), only embryos that retained all three lineages were considered. Treated, n = 6; control, n = 4. Scatterplot, mean ± SEM.

(E) Mouse embryonic stem cells (mESCs) were cultured for 48 h in 3D Matrigel in differentiating conditions. Structures stained for DAPI (red), F-actin (green), and Otx2 (white).

(F) Quantitative analysis of the circularity of mESC structures. n = 40. Scatterplot, mean ± SEM. Structures were collected from 4 independent experiments.

(G) Model for the hypothetical forces required to regulate EPIBLAST cup-shape acquisition. Blue, polar TE; magenta-purple, epiblast. Scale bars, 20 μm.

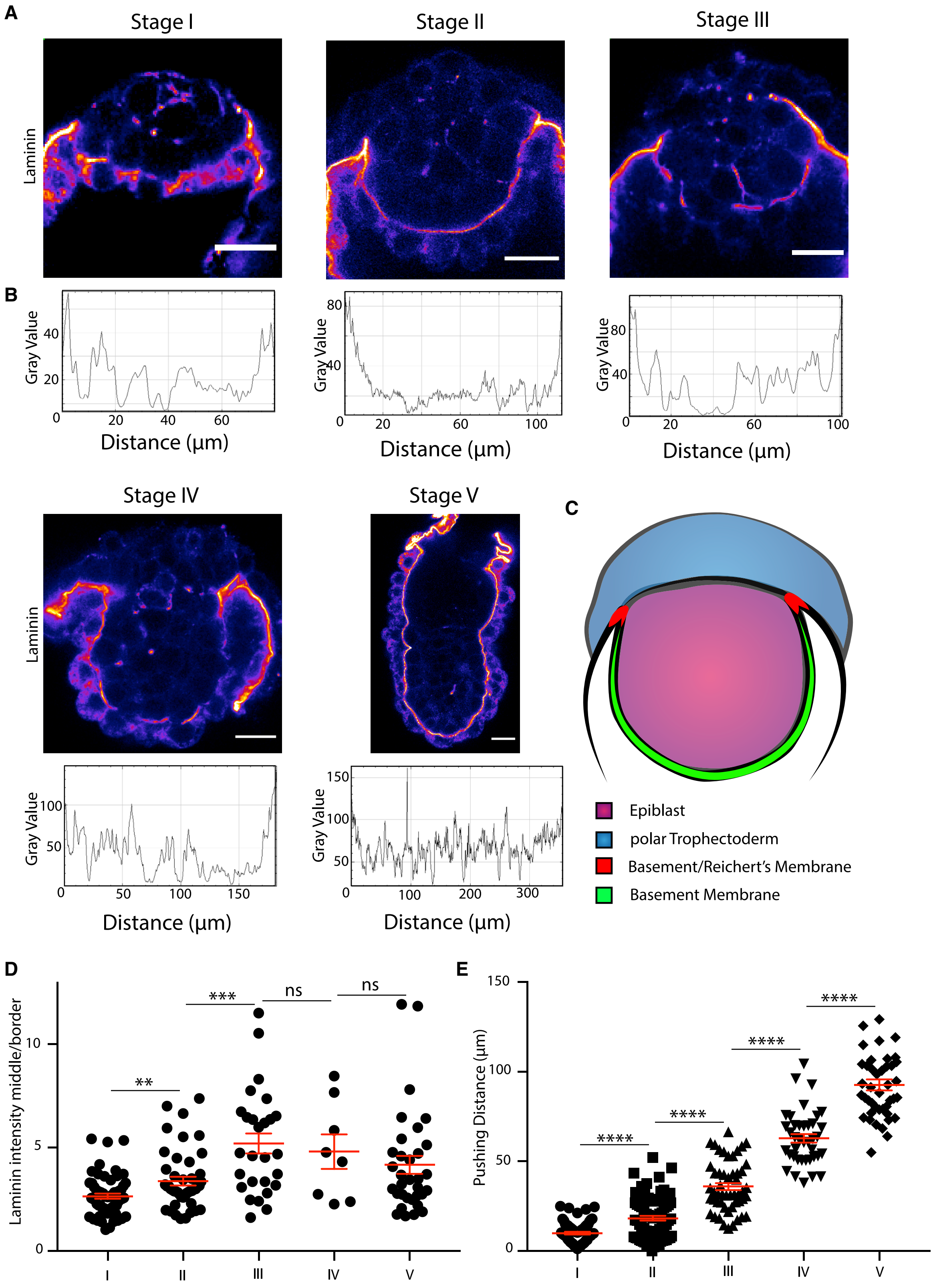

(legend on next page)

CellPress
OPEN ACCESS

To enable the increase of contractility, F-actin must be bound to pMyosin-II. We therefore co-stained embryos for F-actin and pMyosin-II and found that these markers became co-localized increasingly at the apical surface of the polar TE and, with time, spread from being confined to the cell-cell junctions to form a structure reminiscent of supra-cellular actin cables reported in other systems (Galea et al., 2017) (Figures 5B and 5C; Figure S5C). As the polar TE appeared to exhibit contraction across the entire surface of the tissue, we hypothesized that a supra-cellular actin network could generate the force required. To act supra-cellularly, actomyosin must be coupled intercellularly by E-cadherin; therefore, we next examined the expression of E-cadherin, F-actin, and pMyosin-II (Figure 5G; Figure S5D). F-actin and pMyosin-II both became increasingly concentrated toward apical cell-cell junctions, colocalizing with E-cadherin at stage III. At stage IV, a cohesive structure was formed apically, interlinked by E-cadherin and pMyosin-II foci. Surface plots of each factor confirmed this observation, showing a co-localization of all peaks from stage IV onward (Figures 5D and 5H; Figures S5D and S5E). This localization pattern supports the possibility that physical force originating from the polar TE governs epiblast cup-shape acquisition. To investigate whether, indeed, increased tension and contractility in the polar TE could contribute to the regulation of epiblast shape, we treated embryos at E4.5 with Blebbistatin in hanging drops for 20 h to prevent pMyosin-II-mediated contractility. Control embryos developed a total curvature angle of 122° between the epiblast and the polar TE (Figures 5I and 5J), whereas in Blebbistatin-treated embryos, the total curvature was significantly higher (90°). These results suggest that pMyosin-II-mediated contractility in the polar TE leads to shape changes in the epiblast.

Human blastocysts form bilaminar discs upon implantation

To understand if TE behavior could influence epiblast shape, we next focused on human embryos, where the polar TE mediates the implantation process (Figure 6A). The epiblast evolves into a bilaminar disc rather than a cup shape (Hertig et al., 1956). We hypothesized that this could be due to stretching of the epiblast through the polar TE. To investigate epiblast shape upon implantation, we analyzed the embryos of the Carnegie Collection that have been obtained through sectioning of uteri (Hertig, 1945; Hertig et al., 1956). Our observations show that human epiblasts initially form an oval shape, similar to that of mouse embryos, but then become disc-like, growing horizontally but not vertically (Figures 6B–6D). Since the low embryo numbers of the Carnegie Collection do not allow robust quantitative analysis, we analyzed a dataset of 58 pre- and post-implantation *in vitro* cultured human embryos (M.Z-G. et al., unpublished data). These analyses revealed that the epiblast followed a trend similar to that observed in the Carnegie stages, resulting in the acquisition of a flat oval shape during post-implantation stages (Figure 6E). The epiblast circularity decreased significantly upon attachment with a similar value as in the Carnegie stages (Figures 6C and 6F). Similarly, we observed horizontal growth of the epiblast upon implantation in parallel with our observations from the Carnegie stages (Figures 6D and 6G).

As contractile tension appears to drive epiblast remodeling in mouse embryos, we hypothesized that loss of such tension would lead mouse embryos to acquire the disc-like shape of human embryos. To test this, we cultured E4.5 mouse embryos for 20 h with the Rock inhibitor Y27632 (treated) or DMSO (control). We found that Rock inhibition led embryos to lose pMyosin-II expression, indicating that the inhibition was successful (Figure S6A), and that the epiblast of the treated embryos developed into a flat disc or spread over the primitive endoderm in contrast to control embryos, of which 36% established a cup-like shape by the time of fixation (Figures 6H, 6I, and S6B). Thus, the degree of contractile tension in the TE appears to mediate epiblast shape upon implantation.

DISCUSSION

In this study, we provide a comprehensive analysis of the morphogenetic events that lead to tissue remodeling of the mouse embryo upon implantation. We describe how the epiblast and polar TE change their shape over time and provide evidence that epiblast shape is determined by physical force exerted upon it by the overlying TE. We show that the epiblast does not proliferate locally to change from an oval shape into its characteristic cup shape by flattening its interface with the polar TE and growing distally into the blastocoelic cavity. Instead, it transits through five distinct stages, starting off as an oval structure, developing into a spherical and then a rhomboid shape, sharing a highly angled interface with the polar TE. Only then does the EPI transform into a cup (Figure 7).

It has remained unclear how much impact the exit from naive pluripotency has upon epiblast shape. Our results show that exit from naive pluripotency toward the formative state (Smith, 2017) initiated upon implantation is completed before remodeling of the epiblast, in accord with previous studies (Acampora et al., 2016; Shahbazi et al., 2017). Formation of the cup shape does

Figure 4. The ECM shows a clear distribution upon implantation

(A) Expression of laminin upon implantation from stages I–V. Fire staining represents intensity of signal, with purple indicating low intensity and yellow indicating high intensity.

(B) Intensity profiles of laminin signal from (A). BM was traced by spline fit; line width, 5 μm.

(C) Schematic for intensity quantification of laminin expression. Mean gray value of BM (green) was determined through tracing by spline fit, with a line width of 5 μm. Mean gray value of the border between the BM and Reichert's membrane (red) was determined.

(D) Quantitative analysis of laminin intensity ratio BM versus mean of the border between Reichert's membrane and the BM for each embryo. Intensity ratio increases significantly up to stage III and then remains constant. Scatterplot, mean ± SEM. Stage I, n = 59; stage II, n = 46; stage III, n = 27; stage IV, n = 8; stage V, n = 33. Analysis, unpaired Student's t test: stages I–II, p = 0.0023; II–III, p = 0.0002; III–IV, p = 0.6952; IV–V, p = 0.5181.

(E) Quantification of the pushing distance of the epiblast distal tip toward blastocoelic cavity. Epiblast pushed down continuously with a high significance. Mean ± SEM. Stage I, n = 65; stage II, n = 81; stage III, n = 51; stage IV, n = 39; stage V, n = 43. Analysis, unpaired Student's t test: stages I–II, p < 0.0001; II–III, p < 0.0001; III–IV, p < 0.0001; IV–V, p < 0.0001. Scale bars, 20 μm.

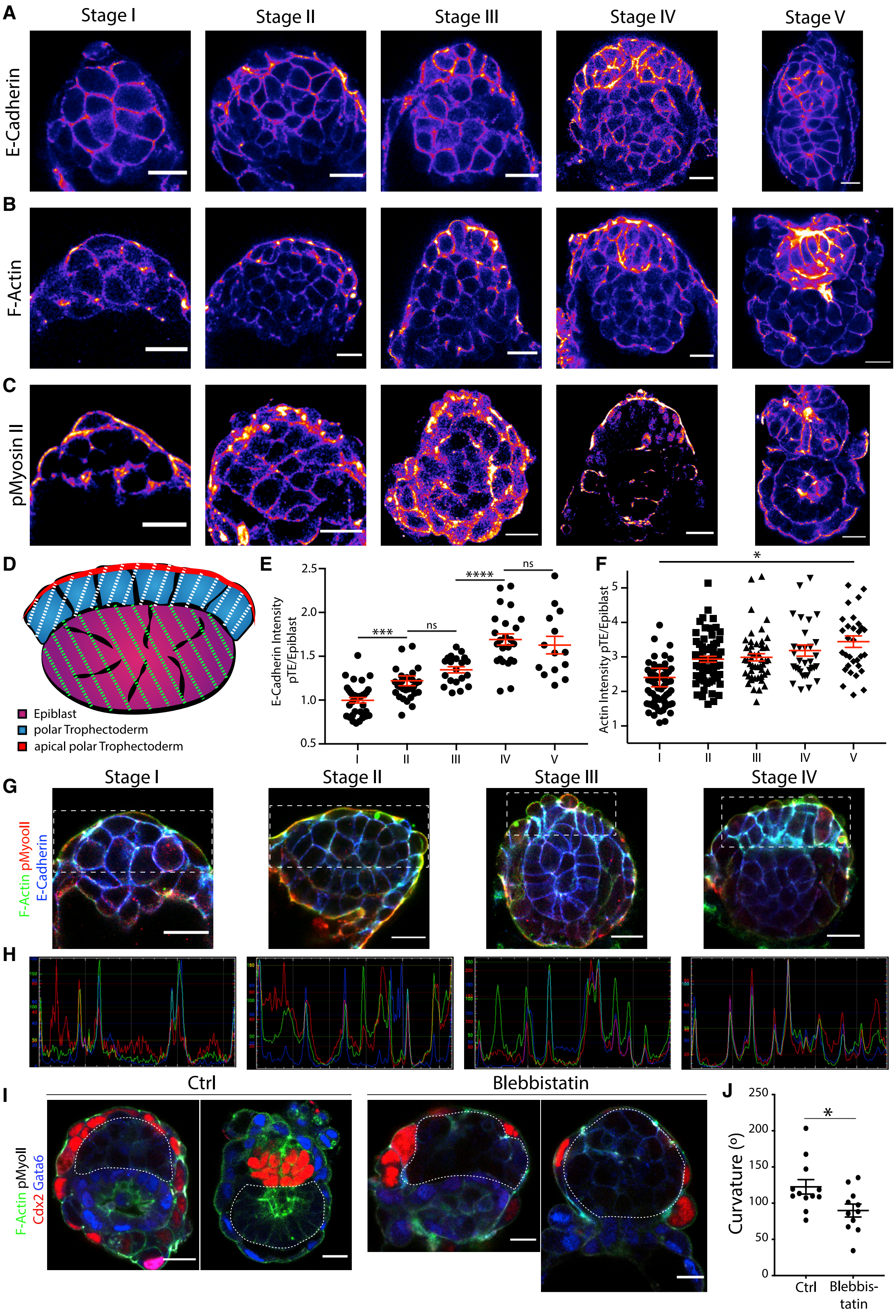

(legend on next page)

not occur autonomously in the epiblast lineage but is induced by the TE. Consequently, removal of this tissue leads the epiblast to become spherical. These results indicate the TE as key regulator of epiblast shape. While we cannot rule out an additional regulatory role of the primitive endoderm, our analysis of the extent to which the epiblast is pushed into the blastocoelic cavity (Figure 4E; Figure S4A) indicates that the primitive endoderm does not prevent epiblast expansion. As such, we conclude that the primitive endoderm is likely to have only minor effects on the epiblast shape.

To understand how the TE exerts force upon the epiblast, we investigated localization and relative concentrations of actomyosin, E-cadherin, integrin β1, and pMyosin-II, known to exhibit increased intensity in contractile tissues (Ciobanasu et al., 2013; le Duc et al., 2010; Harris et al., 2014; Heer and Martin, 2017), in the epiblast versus the polar TE. E-cadherin, F-actin, and pMyosin-II levels continuously increased in the polar TE. This led us to the conclusion that increasing tension emanating from the polar TE regulates epiblast shape. Removal of contractility through inhibitor treatment resulted in embryos exhibiting a significantly higher angled interface with the primitive endoderm. Strikingly, we could observe the formation of a continuous actomyosin structure that is established at stage III in the polar TE. We hypothesize that this structure is a supra-cellular actin network analogous to actin cables described in other systems (Begnaud et al., 2016). This could only be proven through intercellular recoil after laser ablation, but the spherical shape of the embryo at these stages would make such measurements impossible. Since the surface of the polar TE remains highly concave until the supra-cellular actomyosin structure is formed, we hypothesize that tension in the polar TE increases until it reaches a tipping point; up to this point, the growth of the epiblast could exert a higher pressure than the growing tension in the polar TE. As soon as this point is reached, the surface between these two tissues becomes flat, because tension in the polar TE and pressure through tissue growth from the epiblast become equal. After reaching this stage, the E-cadherin levels remain constant. As this acts as a tension sensor and transmitter (Buckley et al., 2014; Lecuit and Yap, 2015), it is possible that the tension then remains constant to prevent further deformation of the epiblast. If the polar TE were to continue to exhibit increase in tension, the tissue interface should become convex.

Epiblast proliferation exerts pressure in every direction. We did not find any indication for localized cell proliferation. However, we were only able to observe vertical, but no horizontal, growth. This suggests that a repressive factor prohibits such growth (Dzamba and DeSimone, 2017). By analyzing the deposition pattern of ECM, we found that the border of the Reichert's membrane forms a tight ring around the epiblast. This ring could introduce the spatial constraint required for successful cup-shape acquisition. Additional factors may also contribute to the restriction of horizontal growth. We have recently shown that, upon implantation, a tissue boundary becomes established between the polar and mural TE (Christodoulou et al., 2019). Tissue boundaries were found to be essential for morphogenesis in various systems (Diaz de la Loza and Thompson, 2017; Fagotto, 2014) and can exert contractile forces similar to those of the supra-cellular actin cables found in wound healing and morphogenesis (Begnaud et al., 2016; Galea et al., 2017; Tipping and Wilson, 2011). However, as a cable-like structure at this boundary is only observed from the cup to the egg cylinder stage (Christodoulou et al., 2019), it seems unlikely that this boundary exerts sufficient force to restrict the epiblast horizontally. We

Figure 5. Differential expression of E-cadherin and F-actin in polar trophectoderm and epiblast

(A) E-cadherin staining of embryos fixed upon implantation up to egg-cylinder formation. Fire staining represents intensity of signal, with purple indicating lowly expressed and yellow indicating highly expressed.

(B) F-actin staining of embryos fixed at consecutive stages from implantation to egg-cylinder formation; intensity is represented through fire staining, as in (A). A clear increase in the actin intensity from stage I to stage V is visible in the polar TE.

(C) pMyosin-II staining of embryos from implantation to egg-cylinder formation. Intensity is represented through fire staining, as in (A). pMyosin-II exhibits similar staining pattern as F-actin.

(D) Schematic illustration of the intensity measurements on tissue level (epiblast is indicated in magenta-purple, and green stripes indicate the area of tissue intensity measurement; polar TE is indicated in blue, and white stripes indicate the area of tissue intensity measurement) and level at which plot profiles were taken (red).

(E) Quantitative analysis of the E-cadherin intensity ratio. For each embryo, the mean gray value of both tissues was determined at 3 different z positions. The mean of the ratio polar TE/epiblast was plotted. As clearly visible, the polar TE intensity increased significantly over time. Scatterplot, mean ± SEM. Stage I, n = 35; stage II, n = 30; stage III, n = 19; stage IV, n = 24; stage V, n = 14. Analysis, unpaired Student's t test: stages I–II, p = 0.0007; II–III, p = 0.0949; II–IV, p < 0.0001; IV–V, p = 0.5729.

(F) Quantitative analysis of the relative F-actin intensity (polar TE/epiblast) over time averaged for each embryo from measurements of the mean gray value of both tissues at 3 different stages. Scatterplot of average values with mean ± SEM. Relative actin intensity clearly increased over time. Stage I, n = 63; stage II, n = 65; stage III, n = 48; stage IV, n = 33; stage V, n = 32. Analysis, unpaired Student's t test: stages I–V, p = 0.0100.

(G) Expression analysis of F-actin (green), pMyosin-II (red), and E-cadherin (blue) in the polar TE from stage I to stage III. White rectangles in the full figures illustrate the zoom-in region.

(H) Merged plot profiles of the apical surface of the polar TE in (G). A spline fit line was drawn with a thickness of 5 μm. Plot profile was determined through Fiji. Staining: F-actin (green), pMyosin-II (red), and E-cadherin (blue). It is visible that, from stages I–IV, the peaks of each marker begin to overlay.

(I) Representative immunofluorescent (IF) stainings of E4.5 embryos cultured for 20 h in hanging-drop culture supplemented with 100 μM Blebbistatin or DMSO in controls. Embryos were stained for F-actin (green), pMyosin-II (white), Cdx2 (red), and Gata6 (blue); the experiment was carried out 4 times. The shape of the EPIBLAST was annotated through a white dotted line.

(J) Quantitative analysis of the total curvature angle of the tissue interface epiblast/polar TE in treated embryos versus controls. Control, n = 12; treated, n = 11. Analysis, unpaired Student's t test: p = 0.0249; treated and control embryos differ significantly.

Scale bars, 20 μm.

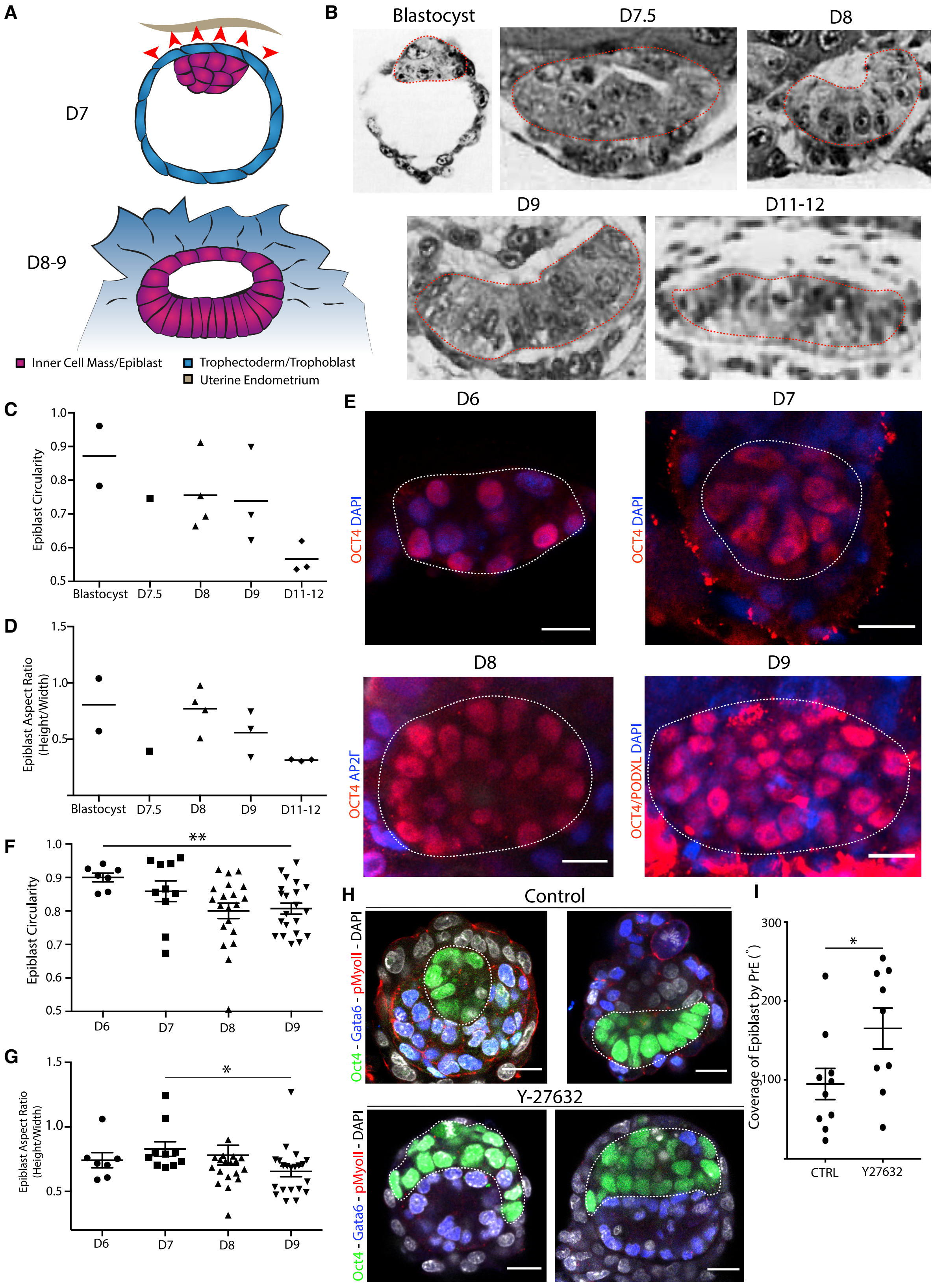

(legend on next page)

therefore conclude that Reichert's membrane is likely to be the key regulator of the epiblast's horizontal growth.

Finally, we also investigated human epiblast morphogenesis that diverges from the mouse during the implantation stages. In contrast to the mouse, the polar TE of the human embryo mediates implantation and invades the maternal endometrium. This should result in pulling and stretching of the embryo. We therefore analyzed embryos that had developed *in vivo* and were dissected at consecutive stages of peri-implantation and early post-implantation development (Hertig et al., 1956; Heuser et al., 1945) or that had developed *in vitro* using a culture method recently established by our lab and others (Deglincerti et al., 2016; Shahbazi et al., 2016). We found that the human embryo does not exhibit any sign of horizontal constraint and develops into a flat oval, the bilaminar disk. To confirm that polar TE stretching alone could shape the epiblast into a disc structure, we cultured mouse embryos in high concentrations of Rock inhibitor allowing attachment and found that this led to development into flat structures. Although Rock inhibition forced the mouse epiblast into a disc-like shape, it is unclear how long the embryo could be maintained in this configuration and whether it could undergo patterning, as these developmental steps might be prohibited through off-target effects of the Rock inhibitor on epiblast and visceral endoderm lineages. To investigate in detail to what end mouse embryos could mimic human morphology, a tissue-specific inducible knockout of Rock or the overexpression of a dominant-negative Rock should be used. Our study provides a precise analysis of tissue-shape remodeling upon implantation in the mouse embryo and shows that epiblast shape is dependent on forces exerted by the TE. Moreover, we demonstrate that species-specific remodeling after implantation is likely due to differences in the behavior of the TE.

STAR★METHODS

Detailed methods are provided in the online version of this paper and include the following:

- KEY RESOURCES TABLE
- RESOURCE AVAILABILITY
  - Lead contact
  - Materials availability
  - Data and code availability
- EXPERIMENTAL MODEL AND SUBJECT DETAILS
  - Mouse embryos
  - Mouse embryonic stem cells
- METHOD DETAILS
  - Mouse embryo recovery
  - Mouse embryo culture
  - Immuno-surgery and hanging drop culture
  - Inhibitor treatments
  - mESC culture
  - Embryo fixation and IF
  - Imaging, image processing and analysis
- QUANTIFICATION AND STATISTICAL ANALYSIS

SUPPLEMENTAL INFORMATION

Supplemental Information can be found online at https://doi.org/10.1016/j.celrep.2020.108655.

ACKNOWLEDGMENTS

We are grateful to D. Glover, M.A. Molè, N. Christodoulou, C. Gantner, M. Shahbazi, H. Mudge, and E. Paluch for feedback. This study has been enabled through grants from the European Research Council (669198), the Wellcome Trust (207415/Z/17/Z), NIH Pioneer Award Fund (DP1 HD104575-01), Open Philanthropy/Silicon Valley Community Foundation, Weston Havens Foundation to M.Z.G. A.W. is supported by the EU Horizon 2020 Marie Sklodowska-Curie actions grant (ImageInLife,721537) to M.Z.G.

AUTHOR CONTRIBUTIONS

A.W. and M.Z.-G. conceived of and conceptualized the project. A.W. designed and carried out all experiments and data analysis. A.W. and M.Z.-G. wrote the manuscript.

DECLARATION OF INTERESTS

The authors declare no competing interests.

Figure 6. Human epiblasts are not constricted horizontally upon implantation

(A) Schematic drawing of a human blastocyst upon implantation at embryonic day (D)7. Implantation into the maternal endometrium (beige) mediated by the polar TE (blue), overlying the epiblast/inner cell mass (magenta-purple). The implantation results in differentiation and invasion of the trophectoderm/ trophoblast, which is hypothesized to exhibit stretching and pulling forces on the epiblast (red arrows). After implantation, the epiblast acquires a bilaminar disc-like structure forming a flat oval (D8–9).

(B) Analysis of epiblast shapes of embryos from the Carnegie Collection. EPIBLAST is indicated with a red dashed line. Blastocyst: Carnegie embryo 8663; D7.5: Carnegie embryo 8020; D8: Carnegie embryo 8155; D9: Carnegie embryo 8004; D11–12: Carnegie embryo 7700.

(C) Quantitative analysis of epiblast circularity of Carnegie embryos from blastocyst to D11–12. Scatterplot and mean.

(D) Quantitative analysis of epiblast aspect ratio (height versus width) of Carnegie embryos from blastocyst stage to D11–12. Scatterplot and mean.

(E) IF staining of *in vitro* cultured embryos from D6 to D9. Red indicates OCT4 (D6–8) and OCT4 + PODXL (D9); blue indicates DAPI (D6–7 and D9) and AP2Γ (D8). Scale bars, 20 μm.

(F) Quantitative analysis of the circularity of *in vitro* cultured embryos from D6 to D9. The circularity continuously decreases as the epiblast becomes more oval shaped. Scatterplot, mean ± SEM. Analysis, unpaired Student's t test: D6–D9, $p = 0.0042$. D6, $n = 7$; D7, $n = 10$; D8, $n = 20$; D9, $n = 21$.

(G) Quantitative analysis of epiblast aspect ratio (height/width) of *in vitro* cultured embryos from (F). Aspect ratio decreases significantly upon implantation. D7–D9, $p = 0.0219$.

(H) Y27632 treatment of mouse embryos in attachment culture for 20 h. Treated embryos were cultured in 100 μM Y27632, and controls were cultured in DMSO. Embryos stained for Oct4 (green), Gata6 (blue), pMyosin-II (red), and DAPI (gray). Experiment was carried out 3 times. Dashed white line encircles epiblast lineage.

(I) Analysis of epiblast coverage angle by the primitive endoderm in Y27632-treated embryos versus controls. The controls are significantly more highly covered than the treated embryos, in which the coverage angle in several cases inverted with epiblast spreading over the primitive endoderm instead. Analysis, unpaired Student's t test: $p = 0.0425$. Controls, $n = 10$; treated, $n = 9$.

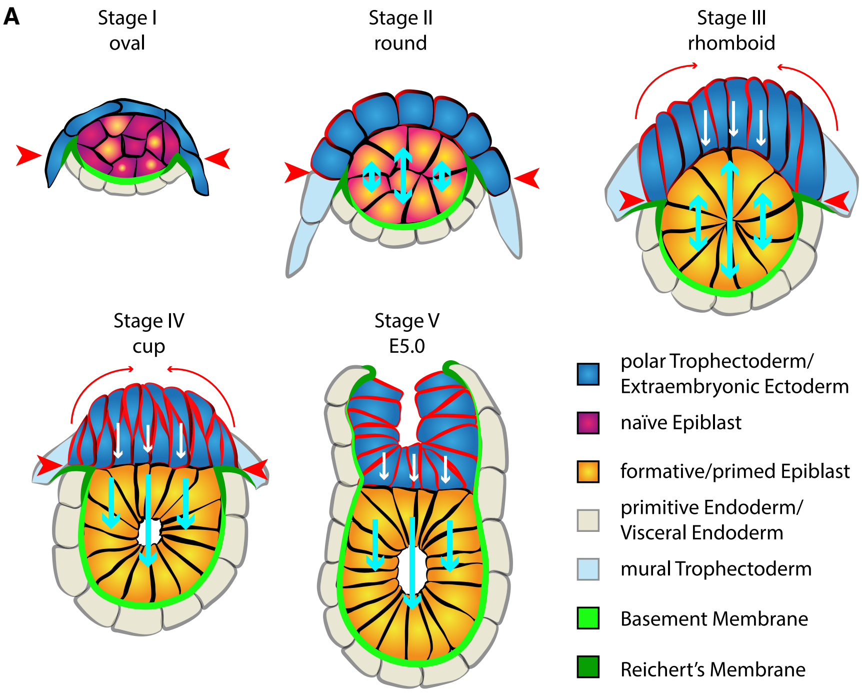

Figure 7. Model of epiblast remodeling at implantation

Upon implantation, stage I, the oval-shaped naive epiblast (magenta-purple) exits from naive pluripotency toward the primed state (yellow-orange) and increases in area. A thick layer of Reichert's membrane constricts horizontal growth (red arrowheads), leading to the epiblast to only grow vertically (light blue arrows) to adopt a circular shape at stage II. At the same time, the polar TE (dark blue) began to increase in height due to a tissue boundary developed toward the mural trophectoderm (light blue) and begins to exhibit increased levels of tension and contractility (red cell membranes). Continuous growth of the epiblast in addition to constrictive force of Reichert's membrane lead to the acquisition of a rhomboid shape (stage III). Then, the polar TE begins to constrict apically (long red arrows), exerting force toward the epiblast, transforming the concave surface to a flat disk, which leads the epiblast to become a cup only able to grow toward the proximal side. Further apical constriction of the polar TE results in formation of the egg cylinder (stage V).

Received: May 8, 2020
Revised: October 12, 2020
Accepted: December 22, 2020
Published: January 19, 2021

REFERENCES

Acampora, D., Omodei, D., Petrosino, G., Garofalo, A., Savarese, M., Nigro, V., Di Giovannantonio, L.G., Mercadante, V., and Simeone, A. (2016). Loss of the Otx2-Binding Site in the Nanog Promoter Affects the Integrity of Embryonic Stem Cell Subtypes and Specification of Inner Cell Mass-Derived Epiblast. Cell Rep. *15*, 2651–2664.

Bedzhov, I., and Zernicka-Goetz, M. (2014). Self-organizing properties of mouse pluripotent cells initiate morphogenesis upon implantation. Cell *156*, 1032–1044.

Begnaud, S., Chen, T., Delacour, D., Mège, R.-M., and Ladoux, B. (2016). Mechanics of epithelial tissues during gap closure. Curr. Opin. Cell Biol. *42*, 52–62.

Buckley, C.D., Tan, J., Anderson, K.L., Hanein, D., Volkmann, N., Weis, W.I., Nelson, W.J., and Dunn, A.R. (2014). The minimal cadherin-catenin complex binds to actin filaments under force. Science *346*, 1254211.

Cartagena-Rivera, A.X., Logue, J.S., Waterman, C.M., and Chadwick, R.S. (2016). Actomyosin Cortical Mechanical Properties in Nonadherent Cells Determined by Atomic Force Microscopy. Biophys. J. *110*, 2528–2539.

Christodoulou, N., Weberling, A., Strathdee, D., Anderson, K.I., Timpson, P., and Zernicka-Goetz, M. (2019). Morphogenesis of extra-embryonic tissues directs the remodelling of the mouse embryo at implantation. Nat. Commun. *10*, 3557.

Chugh, P., Clark, A.G., Smith, M.B., Cassani, D.A.D., Dierkes, K., Ragab, A., Roux, P.P., Charras, G., Salbreux, G., and Paluch, E.K. (2017). Actin cortex architecture regulates cell surface tension. Nat. Cell Biol. *19*, 689–697.

Ciobanasu, C., Faivre, B., and Le Clainche, C. (2013). Integrating actin dynamics, mechanotransduction and integrin activation: the multiple functions of actin binding proteins in focal adhesions. Eur. J. Cell Biol. *92*, 339–348.

Copp, A.J. (1978). Interaction between inner cell mass and trophectoderm of the mouse blastocyst. I. A study of cellular proliferation. J. Embryol. Exp. Morphol. *48*, 109–125.

Copp, A.J. (1979). Interaction between inner cell mass and trophectoderm of the mouse blastocyst. II. The fate of the polar trophectoderm. J. Embryol. Exp. Morphol. *51*, 109–120.

Deglincerti, A., Croft, G.F., Pietila, L.N., Zernicka-Goetz, M., Siggia, E.D., and Brivanlou, A.H. (2016). Self-organization of the in vitro attached human embryo. Nature *533*, 251–254.

Diaz de la Loza, M.C., and Thompson, B.J. (2017). Forces shaping the Drosophila wing. Mech. Dev. *144* (*Pt A*), 23–32.

Dzamba, B.J., and DeSimone, D.W. (2017). Extracellular Matrix (ECM) and the Sculpting of Embryonic Tissues. Curr. Top. Dev. Biol. *130*, 245–274.

Fagotto, F. (2014). The cellular basis of tissue separation. Development *141*, 3303–3318.

Fässler, R., and Meyer, M. (1995). Consequences of lack of beta 1 integrin gene expression in mice. Genes Dev. *9*, 1896–1908.

Galea, G.L., Cho, Y.-J., Galea, G., Molè, M.A., Rolo, A., Savery, D., Moulding, D., Culshaw, L.H., Nikolopoulou, E., Greene, N.D.E., and Copp, A.J. (2017). Biomechanical coupling facilitates spinal neural tube closure in mouse embryos. Proc. Natl. Acad. Sci. USA *114*, E5177–E5186.

Harris, A.R., Daeden, A., and Charras, G.T. (2014). Formation of adherens junctions leads to the emergence of a tissue-level tension in epithelial monolayers. J. Cell Sci. *127*, 2507–2517.

Heer, N.C., and Martin, A.C. (2017). Tension, contraction and tissue morphogenesis. Development *144*, 4249–4260.

Hertig, A.T. (1945). On the development of the amnion and exoccelomic membrane in the previllous human ovum. Yale J. Biol. Med. *18*, 107–115.

Hertig, A.T., Rock, J., and Adams, E.C. (1956). A description of 34 human ova within the first 17 days of development. Am. J. Anat. *98*, 435–493.

Heuser, C.H., Rock, J., and Hertig, A.T. (1945). Two human embryos showing early stages of the definitive yolk sac. Contrib. Embryol., Carnegie Inst. Wash. Publ. *557*, 85–99.

CellPress
OPEN ACCESS

le Duc, Q., Shi, Q., Blonk, I., Sonnenberg, A., Wang, N., Leckband, D., and de Rooij, J. (2010). Vinculin potentiates E-cadherin mechanosensing and is recruited to actin-anchored sites within adherens junctions in a myosin II-dependent manner. J. Cell Biol. *189*, 1107–1115.

Lecuit, T., and Yap, A.S. (2015). E-cadherin junctions as active mechanical integrators in tissue dynamics. Nat. Cell Biol. *17*, 533–539.

Ma, H., Zhai, J., Wan, H., Jiang, X., Wang, X., Wang, L., Xiang, Y., He, X., Zhao, Z.-A., Zhao, B., et al. (2019). In vitro culture of cynomolgus monkey embryos beyond early gastrulation. Science *366*, eaax7890.

Martin, A.C., Gelbart, M., Fernandez-Gonzalez, R., Kaschube, M., and Wieschaus, E.F. (2010). Integration of contractile forces during tissue invagination. J. Cell Biol. *188*, 735–749.

Miner, J.H., Li, C., Mudd, J.L., Go, G., and Sutherland, A.E. (2004). Compositional and structural requirements for laminin and basement membranes during mouse embryo implantation and gastrulation. Development *131*, 2247–2256.

Molè, M.A., Weberling, A., and Zernicka-Goetz, M. (2020). Comparative analysis of human and mouse development: From zygote to pre-gastrulation. Curr. Top. Dev. Biol. *136*, 113–138.

Nelson, W.J. (2008). Regulation of cell-cell adhesion by the cadherin-catenin complex. Biochem. Soc. Trans. *36*, 149–155.

Nichols, J., and Smith, A. (2009). Naive and primed pluripotent states. Cell Stem Cell *4*, 487–492.

Pinheiro, D., and Bellaïche, Y. (2018). Mechanical Force-Driven Adherens Junction Remodeling and Epithelial Dynamics. Dev. Cell *47*, 3–19.

Salamat, M., Miosge, N., and Herken, R. (1995). Development of Reichert's membrane in the early mouse embryo. Anat. Embryol. (Berl.) *192*, 275–281.

Shahbazi, M.N., Jedrusik, A., Vuoristo, S., Recher, G., Hupalowska, A., Bolton, V., Fogarty, N.N.M., Campbell, A., Devito, L., Ilic, D., et al. (2016). Self-organization of the human embryo in the absence of maternal tissues. Nat. Cell Biol. *18*, 700–708.

Shahbazi, M.N., Scialdone, A., Skorupska, N., Weberling, A., Recher, G., Zhu, M., Jedrusik, A., Devito, L.G., Noli, L., Macaulay, I.C., et al. (2017). Pluripotent state transitions coordinate morphogenesis in mouse and human embryos. Nature *552*, 239–243.

Smith, L.J. (1980). Embryonic axis orientation in the mouse and its correlation with blastocyst relationships to the uterus. Part 1. Relationships between 82 hours and 4 1/4 days. J. Embryol. Exp. Morphol. *55*, 257–277.

Smith, A. (2017). Formative pluripotency: the executive phase in a developmental continuum. Development *144*, 365–373.

Smyth, N., Vatansever, H.S., Murray, P., Meyer, M., Frie, C., Paulsson, M., and Edgar, D. (1999). Absence of basement membranes after targeting the LAMC1 gene results in embryonic lethality due to failure of endoderm differentiation. J. Cell Biol. *144*, 151–160.

Solter, D., and Knowles, B.B. (1975). Immunosurgery of mouse blastocyst. Proc. Natl. Acad. Sci. USA *72*, 5099–5102.

Sun, Z., Guo, S.S., and Fässler, R. (2016). Integrin-mediated mechanotransduction. J. Cell Biol. *215*, 445–456.

Svitkina, T.M. (2020). Actin Cell Cortex: Structure and Molecular Organization. Trends Cell Biol. *30*, 556–565.

Tipping, N., and Wilson, D. (2011). Chick amniogenesis is mediated by an actin cable. Anat. Rec. (Hoboken) *294*, 1143–1149.

Wallingford, M.C., Angelo, J.R., and Mager, J. (2013). Morphogenetic analysis of peri-implantation development. Dev. Dyn. *242*, 1110–1120.

Yamada, S., Pokutta, S., Drees, F., Weis, W.I., and Nelson, W.J. (2005). Deconstructing the cadherin-catenin-actin complex. Cell *123*, 889–901.

STAR★METHODS

KEY RESOURCES TABLE

REAGENT or RESOURCE	SOURCE	IDENTIFIER
Antibodies		
mouse-Oct3/4	Santa Cruz	sc-5279; RRID: AB_628051
mouse-Cdx2	Biogenex	MU392-UC; RRID: AB_2335627
rabbit-Cdx2	Abcam	ab76541; RRID: AB_1523334
goat-Gata6	R&D Systems	AF1700; RRID: AB_2108901
rabbit-Nanog	Abcam	ab80892: RRID: AB_2150114
goat-Otx2	R&D Systems	AF1979; RRID: AB_2157172
rabbit-HNF4	Abcam	ab201460
rabbit-Laminin	Sigma Aldrich	L9393; RRID: AB_477163
rat-E-Cadherin	Thermo-Fisher	13-1900; RRID: AB_86571
rabbit-phosphorylated-Myosin II	Cell Signaling Technology	3671S; RRID: AB_330248
rat-GFP	Fine Chemical Products Ltd	GF090R; RRID: AB_10013361
rat-Podxl	R&D Systems	MAB1556; RRID: AB_2166010
rat Integrin β1	Millipore	MAB1997; RRID: AB_2128202
rabbit-cleaved Caspase 3	Cell Signaling Technologies	9664S; RRID: AB_2070042
rabbit phospho Histone 3	Cell Signaling Technologies	9701S; RRID: AB_331535
AF 568 Donkey-Anti-Rabbit	Life Technologies	A10042; RRID: AB_2534017
AF 568 Donkey-Anti Goat	Life Technologies	A-11057; RRID: AB_2534104
AF 568 Donkey-anti-Mouse	Life Technologies	A10037; RRID: AB_2534013
AF 647 Donkey-anti-Rabbit	Life Technologies	A-31573; RRID: AB_2536183
AF 647 Donkey-anti-Goat	Invitrogen	A21447; RRID: AB_141844
AF 488 Donkey-anti-Rat	Life Technologies	A-21208; RRID: AB_141709
Phalloidin-AF 405	Thermo Fisher Scientific	A30104
Phalloidin-AF 488	Thermo Fisher Scientific	A12379
Phalloidin-AF 594	Thermo Fisher Scientific	A12381
DAPI	Thermo Fisher Scientific	D3571
Chemicals, peptides, and recombinant proteins		
B27	Thermo Fisher Scientific	17504001
N2	Home-made - MZG Lab, Thermo Fisher Scientific	17502048
Anti-mouse serum (rabbit)	Sigma Aldrich	M5774
Rat serum (home-made)	Gift of Thorsten Boroviak Lab, Cambridge	N/A
Fibronectin	Sigma Aldrich	FC010
Blebbistatin	Sigma Aldrich	B0560
Y27632	StemCell Technologies, Inc.	72304
PD0325901	Stem Cell Institute, Cambridge	N/A
GSK3 inhibitor	Stem Cell Institute Cambridge	N/A
LIF	Stem Cell Institute Cambridge	N/A
TrypLE Express Enzyme	Thermo Fisher Scientific	12604-021
Matrigel	SLS	354230
Deposited data		
Human embryos (*in vivo*)	Carnegie Collection	https://embryology.med.unsw.edu.au/embryology/index.php/Carnegie_Collection

(*Continued on next page*)

Continued

REAGENT or RESOURCE	SOURCE	IDENTIFIER
Experimental models: cell lines		
Mouse Embryonic Stem Cells	This manuscript	N/A
Experimental models: mice		
CD1 line	Charles River	Strain code: 022
F1 line	Charles River	Strain Code 176
E-Cadherin-GFP homozygous line	Christodoulou et al., 2019	N/A
Software and algorithms		
Fiji	https://imagej.net/Fiji	N/A
GraphPad Prism 7	GraphPad	https://www.graphpad.com/scientific-software/prism/

RESOURCE AVAILABILITY

Lead contact

Requests for resources as well as for further information should be directed to and will be fulfilled the lead contact Dr. Magdalena Zernicka-Goetz (mz205@cam.ac.uk)

Materials availability

This study did not generate new unique reagents.

Data and code availability

The original raw dataset of mouse embryo immunofluorescence images and *in vitro* cultured human embryo immunofluorescence images is available upon reasonable request through the lead contact. The raw data all graphs are based on are found in Table S1.

EXPERIMENTAL MODEL AND SUBJECT DETAILS

Mouse embryos

The mice used were kept according to national and international guidelines in the animal facility. All experiments carried out have been regulated by the Animals (Scientific Procedures) Act 1986 Amendment Regulations 2012 in addition to ethical review by the University of Cambridge Animal Welfare and Ethical Review Body (AWERB). The Home office has authorised the experiments (License number 70/8864). Mice were culled through cervical dislocation upon any identification of a health concern. Males used in this study were between 6 weeks to 11 months old. The females used in this study were between 6-9 weeks old.

Mouse embryonic stem cells

mESCs were derived directly from mouse embryos and have been generated in the MZG lab.

METHOD DETAILS

Mouse embryo recovery

Peri-implantation and early post-implantation stage embryos were dissected from the uteri or deciduas and fixed immediately. Embryos were obtained by crosses of CD1 females with either F1, MF1 or CD1 or endogenous homozygous E-Cadherin-GFP males.

Mouse embryo culture

Embryos were cultured in Advanced IVC medium: CMRL (11530037, Thermo Fisher Scientific) supplemented with 1X B27 (17504001, Thermo Fisher Scientific), 1X N2 (homemade or commercial 17502048, Thermo Fisher Scientific), 1X penicillin–streptomycin (15140122, Thermo Fisher Scientific), 1X GlutaMAX (35050-038, Thermo Fisher Scientific), 1X sodium pyruvate (11360039, Thermo Fisher Scientific), 1X essential amino acids (11130-036, Thermo Fisher Scientific), 1X non-essential amino acids (11140-035, Thermo Fisher Scientific), 1.8 mM glucose (G8644, Sigma). The medium was developed based on an improved mouse culture system (Ma et al., 2019).

Immuno-surgery and hanging drop culture

Immuno-surgery was performed on E4.5 embryos (Solter and Knowles, 1975). Embryos were recovered from mouse uteri and incubated for 15 min in advanced IVC medium supplemented with 20% anti-mouse serum (rabbit, M5774, Sigma Aldrich) for 20 min at

37°C. Following incubation, embryos were washed 3x in advanced IVC medium, placed in IVC medium supplemented with 20% complement (home-made rat serum, gift of Thorsten Boroviak) and incubated for 15 min at 37°C. Embryos were washed for 3x and thereby the trophectoderm lineage, which died through the antiserum and complement incubation, was removed through pipetting. Then, the embryos were placed in placed in hanging drops, 2-2.5 ul of advanced IVC medium supplemented with 30% of FBS and 1ug/ml of Fibronectin (FC010, Sigma Aldrich) for 48h. Hanging drop culture was carried out in order to prevent attachment to the dish and thereby spreading of the epiblast. Each embryo was cultured in a single drop to prevent merging of embryos. After 24h, the embryos were changed to fresh drops.

Inhibitor treatments

Mouse embryos were recovered at E4.5., when implantation was initiated. Following dissection, the mural trophectoderm was removed. For Blebbistatin treatment, the embryos were then cultured for 20h in hanging drops of advanced IVC medium, 30% of FBS and 1ug/ml of Fibronectin (FC010, Sigma Aldrich) supplemented with 100uM of Blebbistatin (B0560, Sigma Aldrich), controls were cultured in DMSO. For Rock inhibition, the embryos were cultured for 20h in advanced IVC medium, 30% of FBS and placed in ibidi dishes to allow attachment (80826, ibidi). Treated embryos were cultured in 100uM of Rock inhibitor Y27632 (72304, StemCell Technologies, Inc.), controls were placed in DMSO.

mESC culture

mESCs were cultured on gelatine (G7765, Sigma Aldrich) in Feeder Cell (FC) medium composed of DMEM (41966, Thermo Fisher Scientific), 15% Fetal Bovine Serum (Stem Cell Institute), 1x penicillin–streptomycin (15140122, Thermo Fisher Scientific), 1x GlutaMAX (35050-038, Thermo Fisher Scientific), 1x non-essential amino acids (11140-035, Thermo Fisher Scientific), 1x sodium pyruvate (11360039, Thermo Fisher Scientific) and 100 μM β-mercaptoethanol (31350-010, Thermo Fisher Scientific), which was supplemented with 2iLIF to preserve naive pluripotency (1 μM MEK inhibitor PD0325901 (Stem Cell Institute), 3 μM GSK3 inhibitor CHIR99021 (Stem Cell Institute) and 10 ng/ml LIF (Stem Cell Institute). mESC propagation was carried out at 37°C, 5% CO_2 in a humidified atmosphere. Medium was changed every 48h. Passaging was carried out every 48-72h depending on confluency and colony size. For this, the cells were washed with phosphate buffered saline (PBS, 10010056, Life Technologies) and then incubated with TrypLE Express Enzyme (12604-021, Thermo Fisher Scientific) for 3 min at 37°C. The reaction was stopped with an excess of FC medium. The cell suspension was centrifuged at 1.000rpm for 5 min and the cells were resuspended in FC-2iLIF medium and seeded in 1:10 or 1:20 dilution.

Cells were trypsinised, resuspended in 1ml of PBS and counted. 15.000 cells/well were seeded in 20 μL of ice-cold Matrigel (354230, SLS) in an ibidi-plate (80821, ibidi) and incubated for 5 min at 37°C. then, cells were incubated for 48h in N2B27 medium to allow differentiation. N2B27 medium was composed of 50% DMEM F12 (21331-020, Thermo Fisher Scientific) and 50% of Neurobasal A (10888-022, Thermo Fisher Scientific). This base was supplemented with 1x B27 (17504001, Thermo Fisher Scientific), 1x N2 (homemade or 17502048, Thermo Fisher Scientific), 100 μM β-mercaptoethanol (31350-010, Thermo Fisher Scientific), 1x penicillin–streptomycin (15140122, Thermo Fisher Scientific) and 1x GlutaMAX (35050-038, Thermo Fisher Scientific).

Embryo fixation and IF

Embryos transferred to 4% paraformaldehyde (PFA) in phosphate buffered saline (PBS) immediately following recovery and kept on ice. Once recovery was completed, embryos were fixed for additional 20 min at room temperature (RT). All incubation steps were carried out in well coated with filtered fetal bovine serum (FBS) to avoid attachment to the bottom of the wells. mESCS were fixed in 4% PFA in PBS for 25 min at RT.

Permeabilisation was carried out through incubation in 0.3% Triton X-100/0.1 M Glycin in PBS for 20 min (Embryos) or 25 min (mESCs). Primary antibody incubation was performed in blocking solution (0.1% Tween-20, 10% FBS, 1% bovine serum albumin (BSA) in PBS) at 4°C overnight. Secondary antibodies and nuclear stain using DAPI (10mg/ml) were prepared simultaneously in blocking solution mixed and centrifuged for 5 min at 14.000rpm. Incubation was carried out for 3h at RT in the dark. Washes were performed in PBS supplemented with 0.1% Tween-20. **Primary antibodies** used: mouse-Oct3/4 (sc-5279, Santa Cruz, 1:200), mouse-Cdx2 (MU392-UC, Biogenex, 1:200), rabbit-Cdx2 (ab76541, Abcam, 1:200), goat-Gata6 (AF1700, R&D, 1:200), rabbit-Nanog (ab80892, Abcam, 1:200), goat-Otx2 (AF1979, R&D Systems, 1:200), rabbit-HNF4 (ab201460, Abcam, 1:1500), rabbit-Laminin (L9393, Sigma Aldrich, 1:300), rat-E-Cadherin (13-1900, Thermo-Fisher, 1:300), rabbit-phosphorylated-Myosin II (3671S, Cell Signaling Technology), rat-GFP (GF090R, Fine Chemical Products Ltd. 1:1000), rat-Podxl (MAB1556, R&D Systems, 1:200), rat Integrin β1 (MAB1997, Millipore, 1:150), rabbit-cleaved Caspase 3 (9664S, Cell Signaling Technologies, 1:200), rabbit phospho Histone 3 (9701S, Cell Signaling Technologies, 1:1000). **Secondary antibodies** used: Phalloidin- Alexa Fluor (AF) 405 (A30104, Thermo Fisher Scientific 1:250), Phalloidin-AF 488 (A12379, Thermo Fisher Scientific, 1:500), Phalloidin-AF 594 (A12381, Thermo Fisher Scientific, 1:250), AF 568 Donkey-Anti-Rabbit (A10042, Life Technologies, 1:500), AF 568 Donkey-Anti Goat (A-11057, Life Technologies), AF 568 Donkey-anti-Mouse (A10037, Life Technologies, 1:500), AF 647 Donkey-anti-Rabbit (A-31573, Life Technologies, 1:500), AF 647 Donkey-anti-Goat (A21447, Invitrogen, 1:500), AF 488 Donkey-anti-Rat (A-21208, Life Technologies, 1:500).

Imaging, image processing and analysis

Imaging of embryos and mESCs was performed on a Leica SP8 confocal microscope using a 63x-oil objective. Z stacks were taken at a step size of 0.6 μm. The images were processed using the Fiji software. Analysis was performed using the Fiji software.

CellPress
OPEN ACCESS

QUANTIFICATION AND STATISTICAL ANALYSIS

For statistical analysis of all quantitative analyses carried out, GraphPad Prism 6.0 was used. The sample size is based on previous experimental experiences. For immuno-surgery, embryos were assigned randomly to either treated or control group, this experiment was carried out 3 times.

For all other quantification, the embryos were collected at least three different dates. Every quantification is shown with single data point clouds in addition to the Mean ± SEM. Each datapoint represents a single embryo.

For intensity measurements of whole tissues, the mean gray value was obtained at three different z-positions per embryo. The mean was used for further analysis. The statistical tests performed on each quantification are annotated in the figure legend.

Cell Reports, Volume *34*

Supplemental Information

Trophectoderm mechanics direct epiblast shape upon embryo implantation

Antonia Weberling and Magdalena Zernicka-Goetz

Supplementary

Supplementary Figure S1

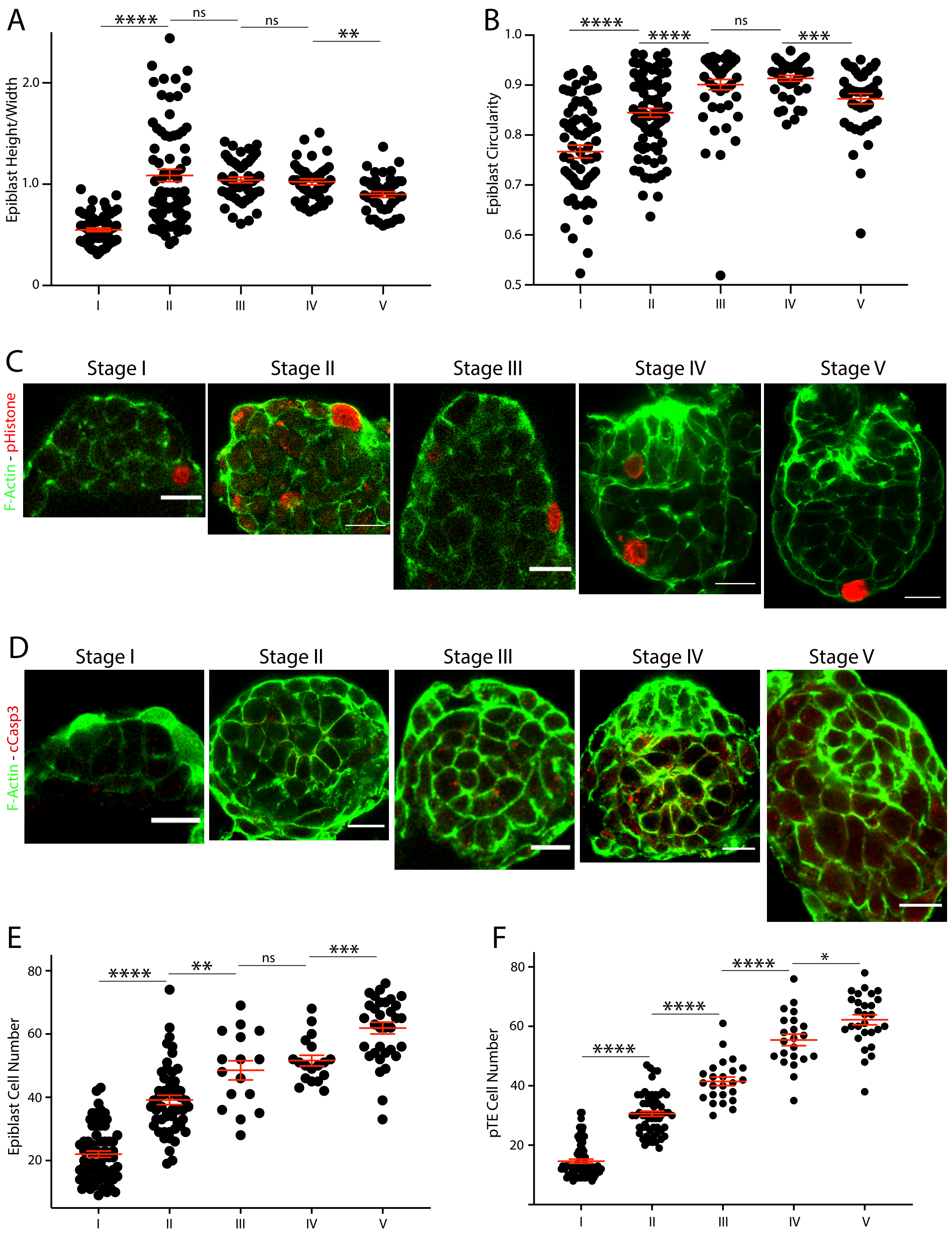

Figure S1. Epiblast and polar trophectoderm do not show polarised cell death or proliferation upon implantation. related to Figure 1 a. Quantitative analysis of epiblast aspect ratio (Height/Width) over time. Scatter plot, Mean±SEM (red). The aspect ratio increased from stage I-II significantly to then stay constant and only decrease slightly from stage IV to V. Analysis: unpaired student's t test. Stage I-II: p<0.0001, stage II-III: p=0.5709, stage III-IV 0.6591, stage IV-V: p= 0.0029. **b.** Quantification of epiblast Circularity over time. Scatter plot, Mean±SEM. The circularity increases first to drop after stage IV. N numbers as in (g). Analysis: unpaired student's t test. Stage I-II: p <0.0001, stage II-III: p <0.0001, stage III-IV 0.3371, stage IV-V: p 0.0012. **c.** IF analysis of pospho-Histone 3 over time. Embryos were stained for F-Actin (green) and phospho-Histone 3 (pHistone) (red). **d.** IF analysis of apoptosis at peri-implantation stages through staining for cleaved Caspase 3 (cCasp3) (red) and F-Actin (green). **e.** Quantitative analysis of epiblast cell numbers over time. The epiblast exhibits a high mitotic index throughout peri-implantation stages. Scatter plot, Mean±SEM (red). Analysis unpaired student's t-test: stage I-II: p<0.0001, stage II-III: p=0.0041, stage III-IV: p=0.3726, stage IV-V: p=0.0007. N numbers: stage I: 71, stage II: 53, stage III: 16, stage IV: 18, stage V: 34. **f.** Quantitative analysis of polar TE cell numbers over time. The polar TE also exhibits a high mitotic index throughout peri-implantation stages. Scatter plot, Mean±SEM (red). Analysis unpaired student's t-test: stage I-II: p<0.0001, stage II-III: p<0.0001, stage III-IV: p<0.0001, stage IV-V: p=0.0114. N numbers: stage I: 71, stage II: 61, stage III: 24, stage IV: 22, stage V: 28.

Supplementary Figure S2

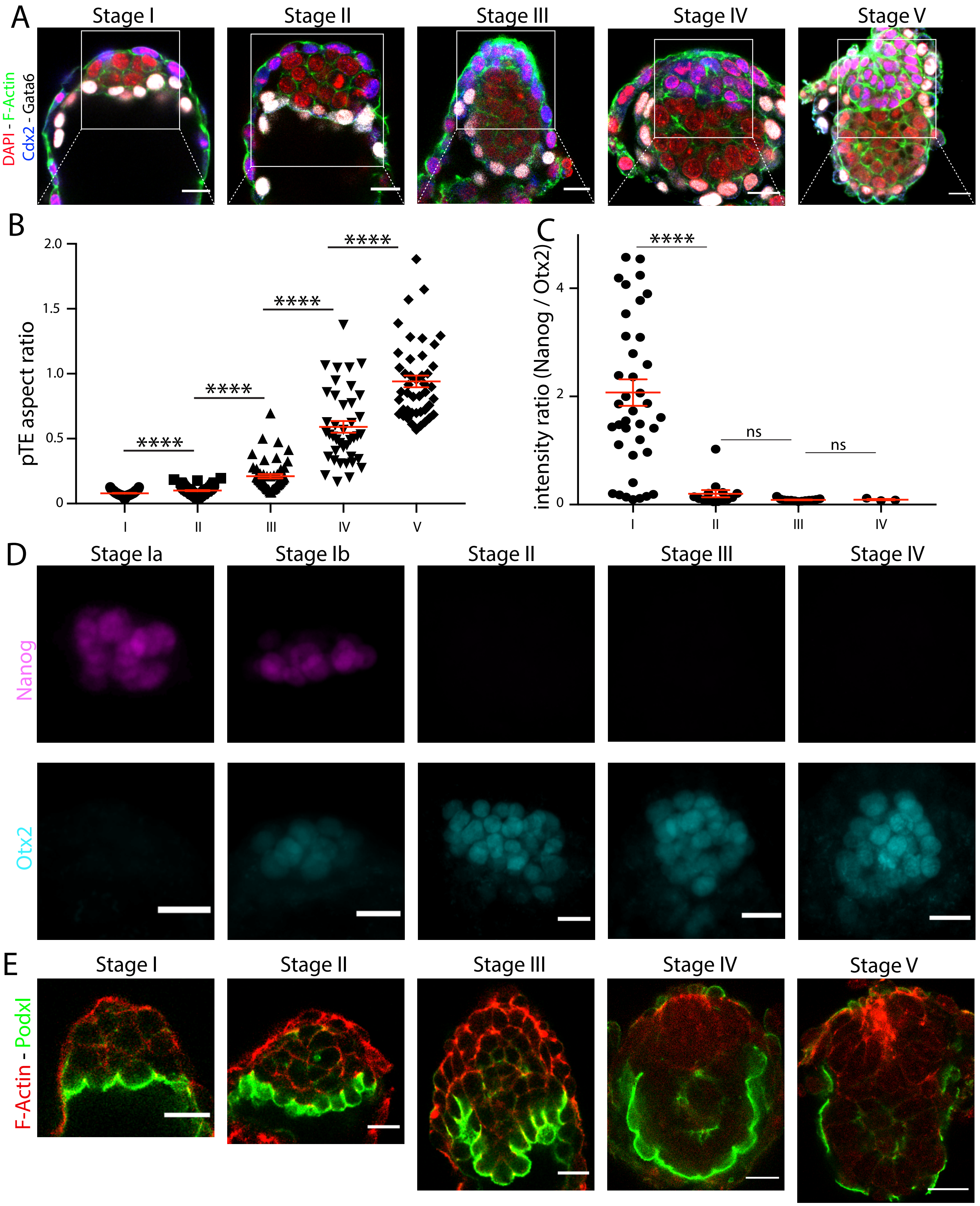

Figure S2. Dynamics of the Tissue interface from implantation to egg cylinder formation, related to Figure 2. a. Lineage staining of embryos fixed at sequential time points from implantation to egg cylinder formation. Full size samples of embryos shown and annotated in Figure 2A. Embryos stained for DAPI (red), Gata6 (white) Cdx2 (blue) and F-Actin (green). **b.** Quantitative analysis of the polar TE aspect ratio (polar TE average height/interface length) over time. Scatter plot, Mean±SEM (red). The aspect ratio increased exponentially. Analysis: unpaired student's t test. Stage I-II: $p<0.0001$, stage II-III: $p<0.0001$, stage III-IV<0.0001, stage IV-V: $p<0.0001$. **c.** Quantitative analysis of the differentiation status of the epiblast. Nanog mean grey value/Otx2 mean grey value. For each embryo, 3 measurements were obtained, the average for each embryo was plotted. The Otx2 expression became clearly upregulated following stage I. In stage I, two clusters are visible. Analysis: unpaired student's t test. Stage I-II: $p<0.0001$, stage II-III: p= 0.1120, stage III-IV 0.7389. **d.** z-projection (Average Intensity) of embryos fixed at consecutive stages following implantation. Nanog was downregulated during stage Ib. Otx2 became upregulated upon stage Ib. **e.** IF Analysis of epiblast polarisation marker PodxI over time. The epiblast becomes fully polarised at stage IV. PodxI (green), F-Actin (red). All scale bars 20µm.

Supplementary Figure S3

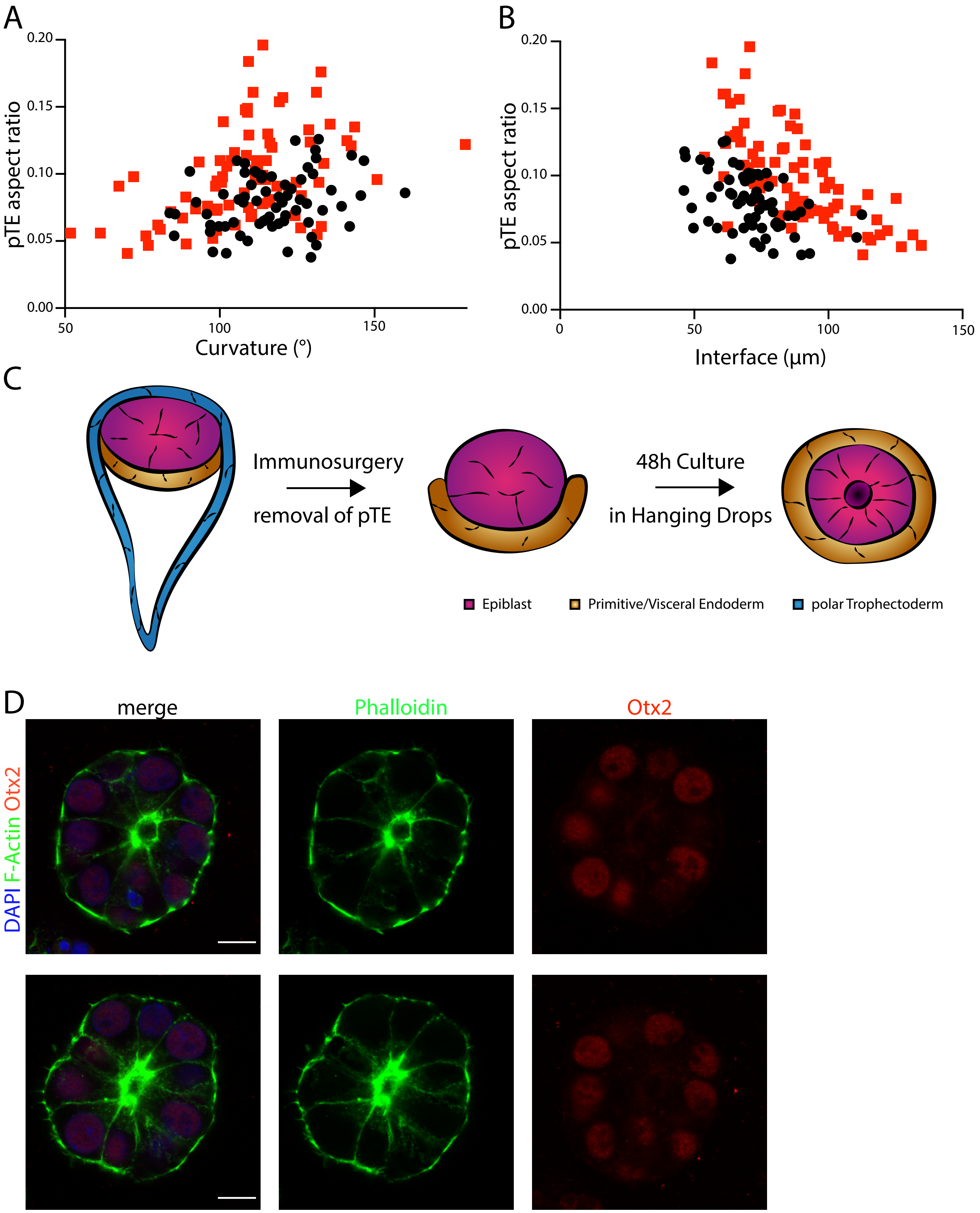

Figure S3. Correlation of polar trophectoderm with curvature and Interface length between stage I and stage II, related to Figure 3. a. Correlation of the polar TE aspect ratio versus the total curvature of the tissue interface for stage I (black) and stage II (red). It is visible, that the two timepoints strongly overly each other. **b.** Correlation of the polar TE aspect ratio versus the total interface length for stage I (black) and stage II (red). It is visible, that these show the opposite trend with an increase in interface length than the following stages (Figure 3B). **c.** Schematic of Immuno-surgery and the following culture. Through Immuno-surgery, the trophectoderm lineage (blue) is removed, the epiblast (red), covered on its' distal side with the primitive endoderm (brown), is cultured in hanging drops for 48h, during which it becomes spherical and opens a lumen while the primitive endoderm spreads to cover the entire epiblast. **d.** Examples of outlier mESC structures grown for 48h in differentiating conditions that exhibited low circularity (Figure 3F).

Supplementary Figure S4

A

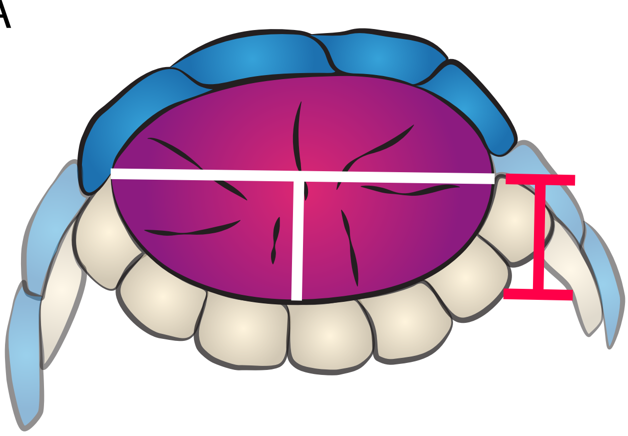

Figure S4. The Epiblast is pushed into the blastocoelic cavity upon implantation, related to Figure 4. a. Schematic of the measurement of the pushing distance. Polar trophectoderm (blue), epiblast (magenta-purple), primitive endoderm (beige). The distance was measured according to the white and red annotations.

Supplementary Figure S5

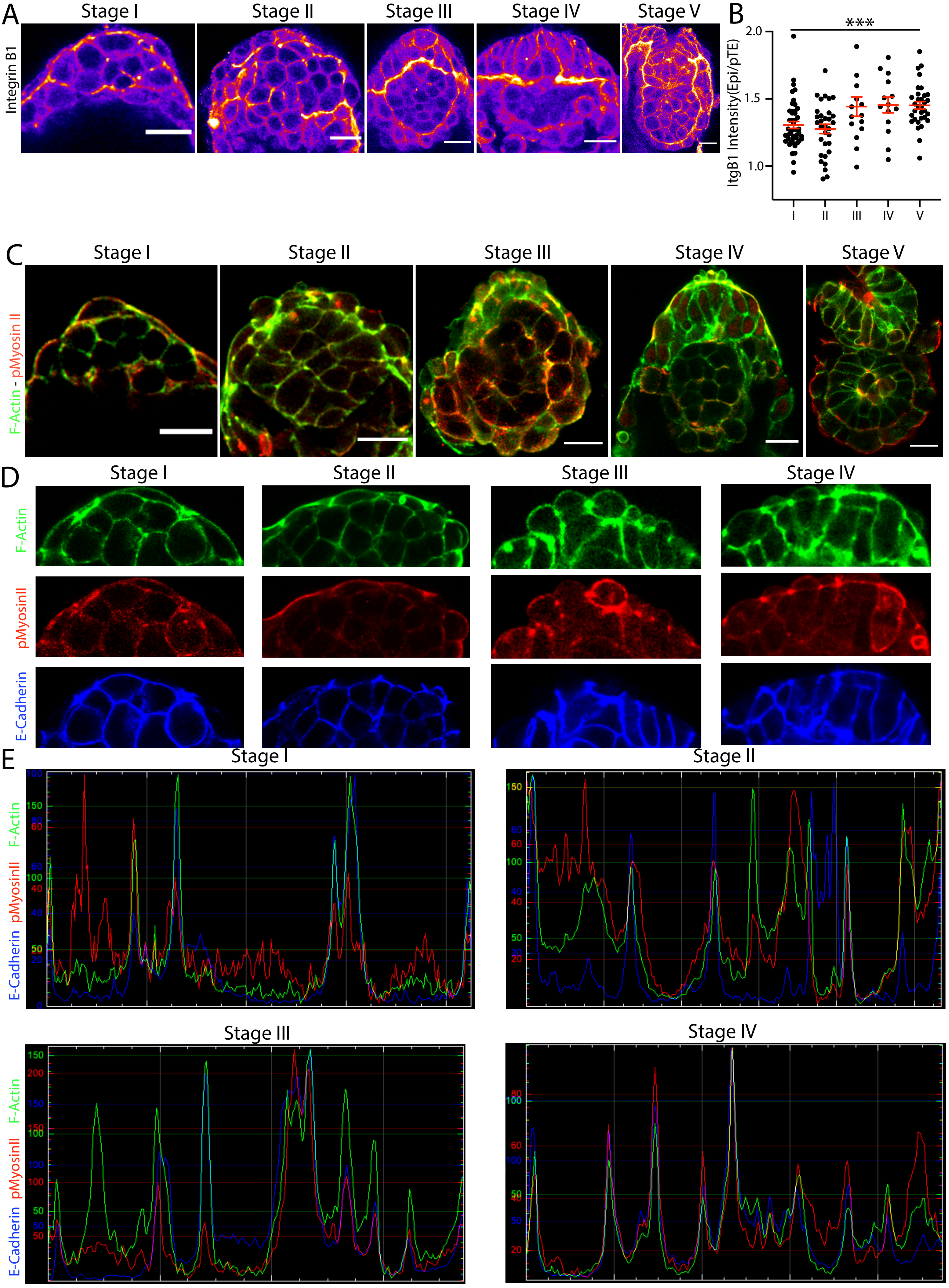

Figure S5. pMyosin II and F-Actin show strong correlation at the apical cell-cell junctions in the polar trophectoderm during epiblast cup shape formation, related to Figure 5. a. IF staining of mouse embryos at peri-implantation stages with basal marker Integrin β1. Fire-staining represents intensity of signal with purple being lowly expressed and yellow showing high signal intensity. It becomes visible, that the polar trophectoderm (polar TE) has a higher intensity of Integrin β1 than the epiblast. **b.** Quantitative Analysis of the intensity of Integrin β1 in epiblast versus polar TE over time. Scatter plot, Mean±SEM (red). Analysis stage I-V: unpaired students t-test: p=0.0004. The intensity increases significantly in the polar TE. N-numbers: stage I: 46, stage II: 32, stage III: 15, stage IV: 14, stage V: 32. **c.** Staining of F-Actin (green) and pMyosin II (red) in embryos fixed at consecutive timepoints upon implantation. Increased localisation of F-actin and pMyosin II becomes visible at the apical cell-cell junctions. From stage III to stage IV, a continuous actin cable could be observed. **d.** Zoom-in of Figure 5g, F-Actin (green), pMyosin II (red), E-Cadherin (blue). **e.** Zoom of Figure 5h: Merged Plot profiles of the apical surface of the polar TE in (c). A spline fit line was drawn with a thickness of 5μm. Plot profile determined through Fiji. Green resembles F-actin, red pMyosin-II, blue E-Cadherin. It is visible that from stage I to stage IV, the peaks of each marker begin to overlay. All scale bars 20μm.

Supplementary Figure S6

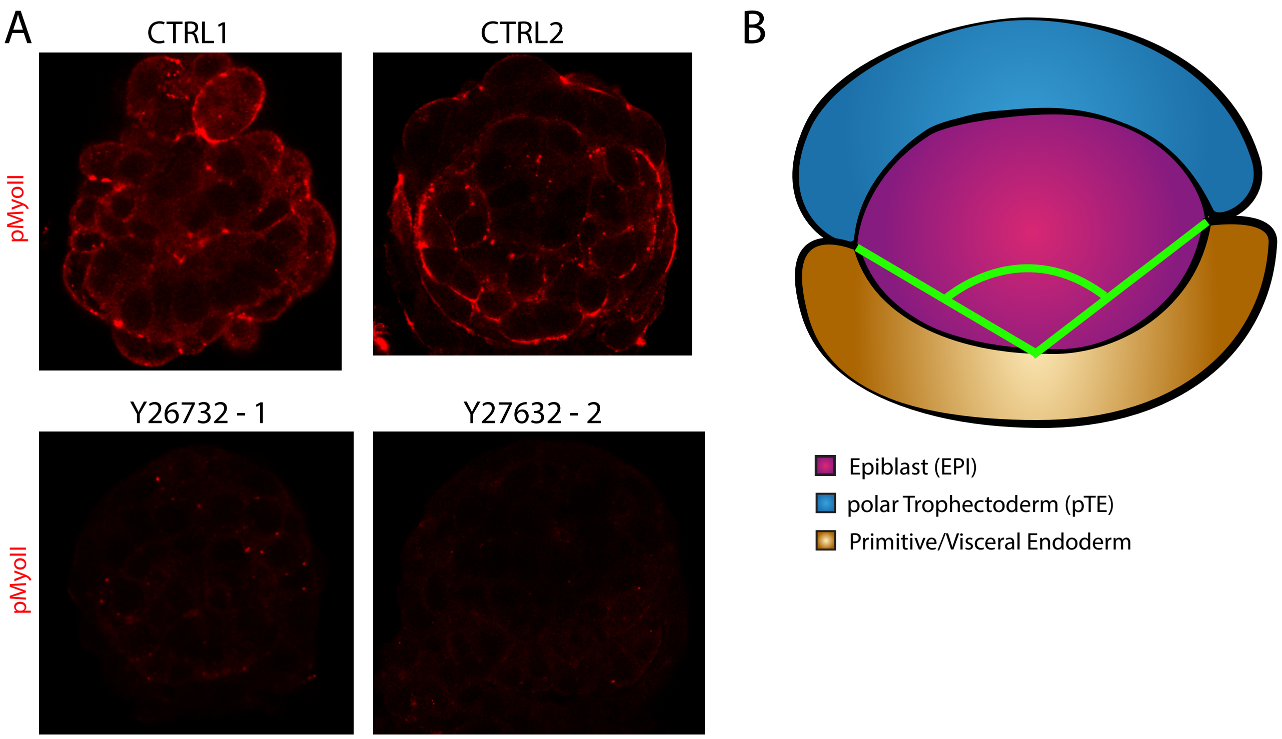

Figure S6: Mouse embryos mimic the human morphogenesis when exposed to Rock inhibitor, related to Figure 6. a. IF staining of pMyosin II channel of the embryos shown in Figure 6h. pMyosin II is lost after Rock inhibition. b. Schematic for quantification of primitive endoderm coverage angle quantified in Figure 6i. Epiblast in red, polar trophectoderm in blue, primitive endoderm in brown. The coverage angle measured is indicated in green.
